# Nutrition Society of New Zealand Annual Conference Held in Wellington, New Zealand, 1–4 December 2015

**DOI:** 10.3390/nu9030239

**Published:** 2017-03-05

**Authors:** Sheila Skeaff, Lisa Te Morenga

**Affiliations:** Department of Human Nutrition, University of Otago, Dunedin 9054, New Zealand; lisa.temorenga@otago.ac.nz

## 1. Preface

The annual conference and scientific meeting of the Nutrition Society of New Zealand took place in Wellington, New Zealand from 1–4 December 2015. Every two years, a joint scientific meeting with the Nutrition Society of Australia is held, alternating between Australia and New Zealand. The 2015 meeting comprised four plenary sessions, 18 concurrent oral sessions and over 100 posters, providing an opportunity for more than 140 nutritional scientists to present their research. Abstracts for plenary talks and for members of the Nutrition Society of New Zealand are published here; abstracts for members of the Nutrition Society of Australia are published in the Journal of Nutrition and Intermediary Metabolism (June 2016). The aim of the combined meeting is to foster discussion and disseminate the results of nutrition-related research undertaken by the members of both societies.

The theme of the 2015 conference was “Past, Present, Future: 100 years of nutrition”, inspired by the 100th anniversary of the ANZAC landing in Gallipoli. The first plenary session was titled ‘Past: Military Nutrition: Optimising soldier health and performance’ utlising speakers that work in national defence including Dr Chris Forbes-Ewan (Australia), Dr James McClung (USA), and Ms Nicola Martin (NZ). The second plenary session was ‘Present: Strategies to address childhood obesity’ and included speakers Dr Beverly Mulhausler (Australia), Associate Professor Louise Signal (NZ), and Professor Rachel Novotny (USA). The third plenary session was titled ‘Present: Emerging concepts of the gastrointestinal tract’ with Professor Gerald Tannock (NZ), Professor Richard Gearry (NZ), and Professor John Pluske (Australia) speaking. The fourth plenary session ‘Future: Nutrients for healthy aging’ included speakers Professor Caryl Nowson (Australia) and Professor Karl-Heinz Wagner (Austria). The Muriel Bell Lecture was given by Professor Marlena Kruger, from Massey University in Palmerston North, NZ, and titled ‘New paradigms for old bones’. In addition to the abstracts found here, a Special Issue of Nutrients titled ‘Selected Papers from 2015 Joint Meeting of the Nutrition Society of New Zealand and Nutrition Society of Australia’ contains seven original research papers and two reviews based on oral or poster presentations and can be found at this URL: http://www.mdpi.com/journal/nutrients/special_issues/2015_joint_scientific_meeting.

## 2. Summary of Scientific Presentations

### 2.1. A Systematic Review of Studies Investigating the Anthelmintic Effect of Datepalm Fruit

Addnan, F.; Mansur, F.A.F.; Manzor, N.F.M.; Rahim Elkadi, M.A.M.A.; Abdullah, W.A.

Background: Datepalm fruit (*Phoenix dactylifera*) is a popular and nutritious food which has been shown to contain many bioactive compounds like carotenoids and phenolics. There is increasing number of scientific reports documenting the health benefits of datepalm fruit. We aim to systematically review the literature for any evidence of the anthelmintic efficacy of datepalm fruit.

Methods: We systematically searched Medline, Ebschohost, ProQuest, Scopus for reports of any studies investigating the anthelmintic effect of datepalm fruit. Search terms included date, palm, phoenix, dactylifera, helminth, anthelmint*. At least two authors independently assessed eligibility of extracted data.

Results: A total of 10 studies were retrieved from the search. However, after further screening the titles and abstracts, only 1 article matched the inclusion criteria for original publication on the anthelmintic effect of datepalm fruit. It was found that datepalm fruit extract had decreased motility of Trichuris muris and Angiostrongylus cantonensis larvae in vitro. Datepalm fruit extract fed to mice infected with Trichuris muris resulted in an arithmetic decrease in faecal egg count during and after treatment. However, these results were not analysed statistically.

Conclusion: Our review revealed the only evidence of the anthelmintic effect of datepalm fruit against parasitic nematodes. The work however, was inconclusive due to lack of statistical analyses and proper experimental design and methodology warranting further systematic and rigorous works to investigate the potential anthelmintic efficacy of datepalm fruit.

### 2.2. A Baseline Survey on Nutrition and Health Claims Used on Supermarket Foods and Beverages in New Zealand

Alexander, D.L.

Background/Aims: The aim of this survey was to assess nutrition and health claims on foods available in New Zealand prior to the full implementation of Standard 1.2.7 Nutrition, Health and Related claims under the Food Standards Code.

Methods: Foods and beverages were randomly selected from a food label database (*n* = 603) and from supermarket visits (*n* = 80), in numbers proportionately represented by 17 categories. Each label was analysed and the presence of any claims, nutrition information and nutrition profile scoring criteria data recorded. All products were assessed for compliance with the requirements of Standard 1.2.7, and reasons for non-compliance recorded.

Results: Nutrient content claims (NCCs) were found on 378 products, with up to 12 claims per label. General Level Health Claims (GLHCs) were on 34 products, with up to eight claims per label. No High Level Health Claims were found. Two products made therapeutic claims. The categories with the highest percentage of NCCs were snack foods, seafood, special purpose foods and dairy products. The highest percentage of GLHCs were on special purpose foods, non-alcoholic beverages and fresh fruits and vegetables. The majority of NCCs were compliant, while the majority of GLHCs were not.

Conclusion: Prior to Standard 1.2.7 coming in to full effect (in January 2016) most manufacturers appear aware of most requirements for NCCs, but not for GLHCs. This baseline data informs the Ministry of Primary Industries of key areas for collaborative work with manufacturers, focusing on specific regulatory requirements, and identifies areas of focus for future compliance surveys.

### 2.3. The Relationship between Dairy Intake, Body Composition, and Physical Activity among Pre-Pubertal Children

Awan, T.; Coad, J.; Poulsen, R.; Brough, L.; Kruger, M.

Background: Milk and dairy products play an important role in optimal growth and development of children.

Objective: To examine dairy intake, body composition including bone mineral status, and physical activity in children.

Methods: A small cross-sectional study of 45 pre-pubertal children (5–10 years). Anthropometric data was collected and physical activity level (PAL) was determined via questionnaire. Body composition and total headless bone mineral content (tBMC), bone mineral density (tBMD), lumbar spine BMC and BMD were measured with dual energy X-ray absorptiometry. Calcium intake was assessed using both food frequency questionnaire-(FFQ) and an estimated 3-day diet record (3DDR).

Results: Average daily serves of dairy (4.8 ± 1.7) and calcium (1474 ± 547 mg) intake were above typical levels. FFQ overestimated calcium intake compared to 3DDR (884 ± 373 mg/day). The mean BMI for boys was 17.4 ± 2.9 and girls was 16.4 ± 1.9 and their respective mean z-scores were +0.53 and +0.11. Boys had significantly higher lean body mass (LBM) (*p* < 0.02) and girls had higher percent body fat (%BF) (*p* < 0.001). tBMC was positively associated with LBM (*p* < 0.001) and total fat mass (TFM) (*p* = 0.004) but negatively to %BF (*p* < 0.0001). tBMD was positively related to LBM (*p* < 0.001) and TFM (*p* < 0.001) but negatively to %BF (*p* = 0.020). PAL was positively associated with tBMD (*p* = 0.013) and negatively with %BF (*p* = 0.003). Calcium/dairy intake was not significantly related to body composition, physical activity, and anthropometric variables.

Conclusion: Dairy consumption in these children was considerably higher than the national average, however high dairy intake did not adversely affect body composition. Body composition and physical activity significantly predicted children’s bone health.

### 2.4. Dietary Protein May Reduce Hospitalisation Due to Infection in Māori of Advanced Age: LiLACS NZ

Baggett, F.; Wham, C.; Teh, R.; Moyes, S.; Kepa, M.; Kerse, N.

Aim: To investigate factors related to hospital admission for infection, examining nutrient intakes of Māori in advanced age (80+ years).

Methods: Face to face interviews with 200 Māori (85 men), in the Bay of Plenty, to obtain demographic, social and health information. Diagnoses were validated against medical records. Detailed nutritional information was collected using the 24 h multiple-pass recall method on two separate days. FOODfiles was used to analyse nutrient intake. National Health Index (NHI) numbers were matched to hospitalisations over a two year period (12 months prior and 12 months following dietary assessment). Infection related hospitalisations were ascertained using International Classification of Disease (ICD) codes.

Results: Participants with infection related hospitalisation had a significantly lower intake of energy-adjusted protein compared to those not hospitalised (15% versus 17% respectively; *p* = 0.009) and a marginally higher consumption of total fat (78.3 g/day vs. 64 g/day; *p* = 0.050) and monounsaturated fat (28 g/day vs. 21 g/day; *p* = 0.040). A total of 18% (*n* = 36) of participants were hospitalised due to infection. The main type of infection was infection of the lower respiratory tract (*n* = 25). Controlling for age, gender, NZ deprivation index, diabetes, CVD and chronic lung disease, a lower energy adjusted protein intake was independently associated with hospitalisation due to infection (OR (95%CI) 1.14 (1.00–1.29), *p* = 0.046).

Conclusion: Protein intake may have a protective effect on the nutrition related morbidity of older Māori. Improving dietary protein intake is a simple strategy to decrease risk.

### 2.5. Dietary Patterns and Socio-Demographic Factors: An Analysis of Data from the 2008/09 New Zealand Adult Nutrition Survey

Beck, K.L.; Jones, B.; Ullah, I.; McNaughton, S.A.; Haslett, S.J.; Stonehouse, W.

Background/Aims: Dietary patterns consider the whole diet rather than individual foods and nutrients. This study aimed to investigate dietary patterns and associations with socio-demographic factors in adult New Zealanders.

Methods: Dietary patterns were identified using factor analysis in adults aged 15 years and over (*n* = 4657) using 24-h diet recall data from the 2008/09 New Zealand (NZ) Adult Nutrition Survey. Multivariate analysis was used to investigate associations between dietary patterns and socio-demographic factors (age, gender, ethnicity, food insecurity, deprivation, education, smoking and supplement use).

Results: Two dietary patterns were identified explaining 12.0% of the variance in food intake. Pattern 1 was a ‘healthy’ dietary pattern characterised by breakfast cereal, low fat milk, yoghurt, bananas, apples, other fruit and tea, and low intakes of pies and pastries, potato chips, white bread, takeaway foods, soft drinks, beer and wine. Pattern 2 was a ‘traditional’ dietary pattern characterised by beef, starchy vegetables, carrots, tomatoes, savoury sauces, regular milk, cream, sugar, tea and coffee, and low in takeaway foods. The ‘healthy’ dietary pattern was positively associated with age, female gender, ethnicity (NZ European or other), never smoking, having a secondary school qualification and taking dietary supplements and was inversely associated with food insecurity and deprivation. The ‘traditional’ dietary pattern was positively associated with age, male gender, smoking (current or ever), food insecurity and inversely associated with having a secondary school qualification.

Conclusion: Dietary patterns were associated with socio-demographic factors. Further research is needed to investigate associations between dietary patterns and nutrition-related risk factors.

### 2.6. Sustained Olive Leaf Phenolic Consumption in Humans and Health Benefits; Transcriptomic Profiling and Mechanisms of Action

Boss, A.; Ferguson, L.; Schlothauer, R.

Background/Aims: Olive leaf extract (OLE) has been used for many years to benefit human health. OLE phenolics have shown therapeutic effects on a range of ailments including; obesity (satiety), cardiovascular disease and cancer. However, the mode of action is not entirely clear. The aim of this study was to identify the genes that respond to OLE to determine underlying mechanisms that correlate to health benefits. A double blind placebo controlled trial design was performed. Secondary aims include studying inflammatory and apoptosis effects of OLE in cell models.

Methods: Gene expression profiles of peripheral blood mononuclear cells from healthy male volunteers (*n* = 29) were analysed using RNA samples for Affymetrix arrays following an 8-week intervention with a 30 mL daily consumption of either OLE or placebo. Difference between groups was determined with one-way ANOVA. Gene expression is being verified by RT-PCR. Nucleotide-binding oligomerization domain-containing protein and HEK-Blue™-2 and 4 cells were analysed for inflammatory response to OLE. Fifty percent survival has been determined for MCF-7 (breast) and LNCap (prostate) cancer cell lines and RT-PCR used to measure differential gene expression.

Results: An interim analysis of the human samples has indicated down-regulated genes that are important in cell proliferation, differentiation, and inflammatory pathways. OLE in cell models demonstrated a strong anti-inflammatory profile in comparison to placebo.

Conclusion: The data in this project supports a gene expression level manipulation by OLE creating health benefits to humans through effects on cell proliferation, differentiation and inflammatory pathways.

### 2.7. Blood Folate Status of NZ Women after a Countrywide Voluntary Programme by the Baking Industry to Fortify Bread with Folate

Bradbury, K.E.; Williams, S.M.; Mann, J.I.; Oey, I.; Aitchison, C.; Parnell, W.; Fleming, L.; Brown, R.C.; Skeaff, C.

Background/Aim: The New Zealand government deferred in 2009, and opted-out in 2012 from a trans-Tasman food standard which mandated that all bread be fortified with folic acid. Instead, in 2010 the government encouraged the New Zealand bread baking industry to implement a voluntary programme to add folic acid to approximately 30% of breads. The aim of this survey was to estimate the effect of the voluntary fortification programme on blood folate status of New Zealand women.

Methods: Women, 18–44 years, living in Wellington (North Island) or Dunedin (South Island) were randomly selected from the Electoral Roll. Participants were asked about consumption of folic acid fortified breads and breakfast cereals. Serum and erythrocyte folate were measured by microbiological assay.

Results: 288 women participated in the survey. Geometric mean (95% CI) serum folate concentration was 30 nmol/L (28–32) and erythrocyte folate was 996 nmol/L (945–1049). These concentrations were 30% to 40% higher compared with women of similar age sampled as part of a national nutrition survey in 2008/09—prior to the voluntary folic acid fortification programme. Reported consumption of fortified bread and fortified breakfast cereal in the past week was associated with 25% (*p =* 0.01) and 15% (*p* = 0.04) higher serum folate concentrations, respectively.

Conclusion: Blood folate status of New Zealand women of childbearing age increased following the introduction in 2010 of the bread baking industry’s voluntary folic acid fortification programme.

### 2.8. Fluoride Intakes in Pregnant Women in Palmerston North, New Zealand

Brough, L.; Jin, Y.; Coad, J.; Weber, J.L.; Thomson, J.A.; Kim, N.

Background: The New Zealand Ministry of Health recommends fluoride is added to public water supplies to a concentration of 0.7–1.0 mg/L. Fluoride is added to municipal water in Palmerston North.

Aims: To assess fluoride intakes among self-selecting pregnant women in Palmerston North.

Methods: Pregnant women (*n* = 59) were recruited from Palmerston North (2009–2011). Daily urinary fluoride excretion (DUFE) was determined by measuring fluoride concentration in 24-h urine samples at an IANZ accredited laboratory (Hill Laboratories, Hamilton) using an ion selective electrode. Total daily fluoride intake (TDFI) was estimated using extrapolation, based on 50% of ingested fluoride being excreted in urine and also using a prediction equation based on healthy adults (TDFI = (DUFE − 0.29)/0.54); pregnancy has little effect on fluoride metabolism.

Results: Median urinary fluoride concentration was 0.82 (0.62, 1.03) µg/mL. Mean DUFE was 1.70 ± 0.78 mg/day. Based on extrapolation, mean TDFI was 3.40 ± 1.56 mg/day; above the AI (3 mg/day) with 26 participants (44%) below the AI. Using the prediction equation, mean TDFI was 2.62 ± 0.91 mg/day; below the AI with 38 participants (64%) below the AI. No participants exceeded the Upper Level (10 mg/day).

Conclusion: Estimates of fluoride intake were lower using the prediction equation than based on extrapolation. The prediction equation suggested inadequate fluoride intakes for these pregnant women. Both methods suggested that toxicity was not a problem as intakes were below the Upper Level. Further research is required to ascertain if fluoride intakes in pregnant New Zealand women are adequate.

### 2.9. Short-Term Effects of Dietary Fibres on Faecal Microbiota and Polysaccharide Content in Rats

Butts, C.; Paturi, G.; Martell, S.; Dinnan, H.; Herath, T.

Background/Aims: Fruit, vegetables and cereals are rich in dietary fibre, vitamins, minerals and phytochemicals. Dietary fibres exhibit prebiotic effects by selectively promoting the growth of beneficial bacteria (*Bifidobacterium* spp., *Lactobacillus* spp.). This study determined the effect of short-term feeding of dietary fibres from cereals, fruit and vegetables on selective faecal bacteria and polysaccharide content using the rat as a model of the mammalian digestive tract.

Methods: Ten-week old rats were fed experimental diets containing 10% cellulose (control), barley β-glucan, broccoli fibre, citrus pectin, inulin, potato fibre, potato resistant starch, or wheat bran, for 7 days. On the final day, faeces were collected from each rat and stored at −80 °C for microbiota and polysaccharide analysis.

Results: The numbers of *Bifidobacterium* spp. were greatest when the rats were fed inulin and potato fibre, while the *Lactobacillus* spp. numbers were enhanced when the rats were given the potato resistant starch and wheat bran diets. Total numbers of faecal bacteria were highest for the rats fed potato resistant starch and lowest for the rats fed citrus pectin. Levels of polysaccharide measured in the faeces was highest for the rats fed cellulose and lowest for the rats fed barley β-glucan, citrus pectin and inulin (*p* < 0.001).

Conclusion: Faecal polysaccharide content reflects the fermentability of these different fibre sources. Consumption of different types of dietary fibre in rats showed differences in faecal microbiota composition, which is likely due to the different abilities/preferences of bacteria for different dietary substrates.

### 2.10. The Relationship between Vitamin D Status and Allergic Diseases in New Zealand Preschool Children

Cairncross, C.T.; Grant, C.C.; Stonehouse, W.; Conlon, C.A.; McDonald, B.; Houghton, L.A.; Eyles, D.; Camargo, C.; Coad, J.; von Hurst, P.R.

Background/Aims: Historically, research on vitamin D deficiency in young children focussed on bone development and rickets. Growing awareness of the immunomodulatory effects of vitamin D has led to the investigation of the relations of vitamin D status to many allergic diseases. Our objective was to investigate this topic in preschool-aged children in NZ.

Methods: Dried capillary blood spots were collected from 1329 children during late winter to early spring for 25(OH)D measurement by LC-MS/MS. Caregivers completed a questionnaire about their child’s recent medical history. Modules of the validated International Study of Asthma and Allergies in Childhood questionnaire were used to identify eczema, allergic rhinoconjunctivitis (ARC) and asthma; diagnosis of doctor-diagnosed food allergy was by parental report. Analysis was by multivariable logistic regression.

Results: Mean 25(OH)D concentration was 52 (SD 19) nmol/L, with 7% of children <25 nmol/L and 49% <50 nmol/L. Children with 25(OH)D concentrations ≥75 nmol/L (*n* = 29) had a two-fold increased risk for parent-report of doctor-diagnosed food allergy compared to children with 25(OH)D 50–74 nmol/L (OR = 2.21, 1.33–3.68, *p* = 0.002). There was a non-significant U-shaped association between 25(OH)D and ARC, with a nadir at approximately 60 nmol/L. No associations were present between 25(OH)D concentration and presence of parent-reported eczema or asthma.

Conclusion: Vitamin D deficiency was not associated with several allergic diseases in these NZ preschool children. In contrast, high 25(OH)D concentrations were associated with a two-fold increased risk of food allergy, with a trend of higher risk of ARC following a U-shaped association with 25(OH)D.

### 2.11. Can Questionnaire-Based Tools to Predict Vitamin D Deficiency Replace Blood Testing for New Zealand Preschool Children?

Cairncross, C.; McDonald, B.; Stonehouse, W.; Conlon, C.A.; Grant, C.C.; Houghton, L.A.; Eyles, D.; Camargo Jnr, C.; Coad, J.; von Hurst, P.R.

Background/Aims: Timely identification and treatment of children with vitamin D deficiency is important. However, vitamin D status determined through serum 25(OH)D sampling can be painful for young children and is not routinely funded in New Zealand. Predictive questionnaire-based tools offer a non-invasive alternative. Our objective was to develop a questionnaire-based tool to predict vitamin D deficiency in NZ preschool-aged children.

Methods: Dried capillary blood spots were collected from 1329 children during late winter to early spring for 25(OH)D measurement by LC-MS/MS. Caregivers completed a questionnaire describing their child’s demographics and factors known to affect vitamin D status. Predictors of 25(OH)D < 25 nmol/L and 25(OH)D < 50 nmol/L were identified using multivariable logistic regression in a randomly-selected sub-sample (*n* = 929) for development of two predictive tools, which then were validated by receiver operating characteristics (ROC) analysis (*n* = 400).

Results: Serum 25(OH)D levels were <25 nmol/L in 7% of children and <50 nmol/L in 49%. The tool predicting 25(OH)D < 25 nmol/L had a sensitivity of 42%, specificity of 97% and AUC of 0.76 (95% CI 0.67–0.86, *p* < 0.001). The tool predicting 25(OH)D < 50 nmol/L had a sensitivity of 52%, specificity of 66% and AUC of 0.63 (95% CI 0.57–0.68, *p* < 0.001).

Conclusion: Despite the large sample size, the questionnaire-based tools were unable to accurately predict vitamin D deficiency in these preschool-aged children, suggesting that blood 25(OH)D testing be used to determine vitamin D status. Further research into safe and inexpensive methods of assessing vitamin D status in vulnerable populations is indicated.

### 2.12. Lean Mass Significantly Predicts Bone Health in Pre-Menopausal Pacific Island Women Living in New Zealand

Casale, M.; von Hurst P.R.; Shultz, S.; O'Brien, W.; Conlon, C.; Beck, K.; Stonehouse, W.; Kruger, R.

Background/Aim: Anecdotally it is suggested that Pasifika women have good bone mineral density (BMD); however, little evidence for this or for associated factors exists. The aim of this study is to explore associations between several key predictors of bone health with bone mineral density, as measured by Z-scores, in pre-menopausal Pasifika women.

Methods: Healthy pre-menopausal Pasifika women (*n* = 91; age 16–45 years) were recruited. Participants’ body composition and total body BMD were assessed using DEXA. A food frequency questionnaire (FFQ) and current bone-specific physical activity questionnaire (cBPAQ) were completed. Variables that significantly correlated with BMD Z-score were applied to a hierarchical multiple regression analysis.

Results: The mean BMD was 1.1 ± 0.08 g/cm^2^. Lean mass (LM, 56 ± 9.4 kg) and total mass (91 ± 20 kg) were the only factors to show a significant correlation with BMD Z-score. Body-fat (38.5% ± 7.5%), cBPAQ score (1.7 (0.4, 5.2)), and dietary calcium (1097 ± 478 mg), protein (17.7% ± 3.5%) and vitamin C (135 mg (97, 233)) showed no correlation with BMD Z-score. The regression analysis suggests LM is the most important predictor of BMD Z-score, explaining 11.5% of the variance, while total mass accounts for a further 3.1% of variance. Together, these factors explain a total of 14.6% of the variability.

Conclusion: LM is the strongest predictor of BMD, while many established contributors to bone health (calcium, physical activity, protein, and vitamin C) do not appear to be associated with BMD in this population. As just 14.6% of the variability can be explained, further research is needed in this area.

### 2.13. Marginal Ascorbate Status (Hypovitaminosis C) Results in an Attenuated Response to Supplementation

Carr, A.; Pullar, J.; Bozonet, S.; Vissers, M.

Background/Aims: Inadequate dietary intake of vitamin C results in hypovitaminosis C, i.e., a plasma ascorbate concentration ≤23 µmol/L. The current Australasia recommended dietary intake (RDI) for vitamin C is 45 mg/day which may be insufficient for such individuals. Our aim was to carry out retrospective analysis of two vitamin C supplementation studies to determine whether supplementation with the current RDI is sufficient to restore adequate ascorbate status (≥50 µmol/L) in individuals with hypovitaminosis C.

Methods: Plasma ascorbate data from 70 young adult males, supplemented with 50 or 200 mg/day vitamin C for 4–6 weeks, was pooled and analysed. Hypovitaminosis C individuals were identified based on their plasma ascorbate status being ≤23 µmol/L. Total dietary intake data from 80% of the participants was also analysed.

Results: Participants consuming 50 mg/day vitamin C had plasma concentrations of ~50 µmol/L within four weeks, whereas those with hypovitaminosis C reached only ~30 µmol/L, despite comparable dietary intakes. Participants consuming 200 mg/day vitamin C reached saturating concentrations (>65 µmol/L) within one week, while those with hypovitaminosis C required two weeks to reach saturation. Regression modelling indicated that the participants’ initial ascorbate status and body weight explained ~30% of the variability in the final ascorbate concentration.

Conclusion: Our analysis reveals that vitamin C supplementation with equivalent to the current Australasian RDI is insufficient to achieve adequate plasma ascorbate concentrations in individuals with hypovitaminosis C. This indicates that dietary intakes greater than the RDI are required to meet their health needs.

### 2.14. The Hypoglycemic Potential of Antioxidant-Rich Foods

Chepulis, L.

Background/Aims: Obesity and diabetes are highly prevalent in NZ and other Western countries, and both conditions are linked with impaired glucose control. Antioxidants have been identified as having the ability to regulate plasma glucose levels, but the effects of specific high-antioxidant foods on plasma glucose levels have not been well characterised.

Methods: Ten healthy individuals were recruited into a non-randomised, non-blinded pilot study. Participants were asked to consume various high-antioxidant foods 10 min prior to 50 g of available carbohydrate from either glucose or white bread after an overnight fast. All antioxidant foods were matched for total antioxidant content. Blood glucose levels were measured using capillary sampling every 15 min for two hours, and the area under the glucose curve (AUC) measured. The AUC values for the test foods were compared to the glucose-only and bread-only controls.

Results: Green Tea and Propolis were both strong glycemic modulators, significantly decreasing the AUC by 25%–31% compared to the glucose-only or white bread controls (all *p* < 0.05). Blueberries significantly decreased the AUC by 19% compared to the glucose control, but was not significantly different to the bread control.

Conclusions: Certain high antioxidant foods are able to reduce the AUC of glucose and white bread; thus they may offer a potential means by which glycemic control could be managed in individuals who have impaired glucose control.

### 2.15. Impact of a Baby-Led Approach to Complementary Feeding on Iron Status at 12 Months of Age: A Randomised Controlled Trial

Daniels, L.; Taylor, R.W.; Gibson, R.S.; Samman, S.; Haszard, J.J.; Williams, S.M.; Wheeler, B.J.; Taylor, B.J.; Heath, A.-L.M.

Background/Aims: The Baby-Led Introduction to SolidS (BLISS) study is a randomised controlled trial of a version of baby-led weaning (BLW) modified to address concerns including a proposed increased risk of iron deficiency. In BLISS and BLW the baby is given foods they pick up and feed themselves. The aim was to determine the extent to which BLISS prevents iron deficiency at 12 months of age.

Methods: 206 pregnant women were randomised into one of two groups: Usual care or BLISS (milk-feeding support from a lactation consultant from late pregnancy to 6 months, then advice from research staff on introducing “solids” using a baby-led approach). BLISS parents were encouraged to offer a high-iron food (red meat or iron-fortified infant cereal) at every meal. Venous blood was collected at 12 months to determine plasma ferritin (PF), soluble transferrin receptor, haemoglobin, C-reactive protein (CRP) and α_1_-acid-glycoprotein (AGP). PF was adjusted if CRP or AGP were elevated. Body iron was calculated and iron deficiency anaemia (IDA), iron deficient erythropoiesis (IDE) and iron depletion (ID) determined.

Results: There was no significant difference in geometric mean PF concentration between BLISS (*n* = 60; 26.3 μg/L) and Usual care (*n* = 59; 28.2 μg/L) groups (difference: −6.8% [95% CI: −27.4%, 19.7%]; *p =* 0.58). Nor were there differences in body iron (difference: 0.2 mg/kg; 95% CI: −0.9, 1.3; *p =* 0.72) or prevalence of IDA, IDE or ID (all *p* ≥ 0.56).

Conclusion: Advice to frequently offer iron-rich foods may prevent the increased risk of iron deficiency expected to be associated with a baby-led approach to complementary feeding.

### 2.16. Motivation, Self-Determination and 5-Year Weight Change in a Population of New Zealand Mid-Life Women

Davidson, R.; Haszard, J.; Leong, S.L.; Horwath, C.

Background/Aim: The aim of this study was to examine in New Zealand mid-life women the relationship between different forms of motivation (as specified by Self-Determination Theory) and change in BMI over 5 years. A secondary aim was to explore potential mediators of this relationship.

Methods: In May 2014, self-administered questionnaires were sent to participants in a nationwide longitudinal study (baseline: 2009, 2-years: 2011, 3-years: 2012). Multiple regression was used to explore the relationship between different forms of motivation at baseline assessed using the Regulation of Eating Behaviour Scale (REBS) and change in BMI over 5-years. Mediation analysis was conducted using the approach of Sobel-Goodman.

Results: At 5-year follow-up, 899 questionnaires were returned (response rate = 73%, mean age 50.6 years). Regarding REBS scores at baseline and change in BMI over 5-years, a 12-point higher score for baseline autonomous regulation was associated with a 0.24 kg/m^2^ decrease in BMI over 5-years (95% CI: −0.36, −0.12, *p* = 0.001). In contrast, a 4-point higher baseline amotivation score was associated with a 0.20 kg/m^2^ increase in BMI over 5-years (95% CI: 0.04, 0.40, *p* = 0.020). Potential mediators identified were: variety of vegetables consumed, consumption of high energy density foods and intuitive eating. However, no significant mediators were found for the relationship between baseline REBS scores and change in BMI over 5-years.

Conclusion: This study suggests that autonomy supportive approaches to promoting healthy eating may be helpful in preventing an increase in BMI over time.

### 2.17. How Do the Eating Habits and Diet Quality of Pacific Youth Living in New Zealand Contribute to Their Risk of Developing Obesity?

Deo, N.N.; Kruger, R.; Breier, B.H.; Kaholokula, K.; Foliaki, S.; O’Connell, A.; Borman, B.; Ellison-Loschmann, L.; Firestone, R.

Background: Pacific youth (aged 16–24 years) living in New Zealand (NZ) are at high risk of developing obesity. As part of an over-arching cross-sectional study, we investigated eating habits and diet quality of Pacific youth.

Design: Structured questionnaires were developed and implemented (*n* = 30) using face-to-face interviews. Eating habits, meal patterns, food choices and related cultural and social influences were explored qualitatively. Dietary diversity (food groups) and variety (foods) were explored using a Pacific-focused dietary diversity questionnaire. Eating habits data were analysed using a content analysis approach reflecting meal patterns and food consumption trends. A scoring method was used to capture dietary diversity scores (DDS—26 food groups: 15 nutritious; 11 discretionary) and food variety scores (FVS—227 foods: 129 nutritious; 98 discretionary).

Outcomes: A two-meals/day eating pattern was observed; breakfast skipping was apparent. Snacking frequency was higher amongst meal skippers. High availability of discretionary foods, cost, convenience and cultural values around food consumption all contributed to poor food choice and overeating at social occasions. The mean total, nutritious and discretionary DDS over seven days was 23.1, 14.3 and 8.83 groups respectively and FVS was 91, 51.7 and 39.3 foods, respectively.

Conclusion: Eating habit assessment identified the barriers experienced by Pacific youth influencing their current diet quality. Despite a large dietary variety, nutritious foods contributed only moderately to diet quality, whilst discretionary foods expanded dietary energy density. These observations explain, at least in part, the increased risk of developing obesity and could guide future interventions.

### 2.18. Dietary Intakes and Food Sources of Long Chain Omega-3 Polyunsaturated Fatty Acids of Pregnant Women Living in New Zealand

Eickstaedt, M.; Beck, K.L.; Conlon, C.A.

Background/Aims: Adequate intakes of Long Chain Omega-3 Polyunsaturated Fatty Acids (n-3 LC-PUFAs) are required for foetal growth, brain development and to support a healthy pregnancy. This study aimed to investigate dietary intakes and food sources of n-3 LC-PUFAs (DHA and EPA) in a cohort of New Zealand (NZ) pregnant women.

Method: Pregnant women (*n* = 596) in their 3rd trimester of pregnancy from throughout NZ completed an online validated FFQ to assess PUFA intakes over the past three months. PUFA intakes were compared with dietary recommendations using frequency summary statistics and one-sampled *t*-tests.

Results: Estimated mean ± SD daily intakes were: 360 ± 510 mg total n-3 LC-PUFA (recommended 500 mg/day; *p* ˂ 0.001), 160 ± 260 mg EPA (recommended 220 mg/day; *p* ˂ 0.001), and 200 ± 250 mg/day DHA (recommended 200 mg/day; *p* = 0.87), with 30.9% of participants consuming more than 200 mg/day DHA. Participants taking PUFA supplements (19.6%) had mean intakes of 430 ± 310 mg/day DHA, with 79.5% meeting DHA recommendations. For participants not taking PUFA supplements (80.4%), DHA intakes were 140 ± 200 mg/day and only 19% met the recommendations. Across all women fish/seafood were the main contributors of DHA (84.8%) and EPA (82.1%) intakes, yet only 9.5% and 12.2% of women consumed canned fish or fresh/frozen fish at least twice per week.

Conclusion: The majority of women did not meet the recommended intakes for DHA, which may be in part due to low fish/seafood intakes. These findings highlight the need for improved nutrition advice on the benefits of consuming n-3 LC-PUFA rich foods such as fish/seafood during pregnancy.

### 2.19. Medium Chain Triglyceride Oil (MCT Oil), a Placebo with Unexpected Adverse Effects

Ferguson, L.; Laing, B.; Marlow, G.; Jesuthasan, A.; Agnew, M.; Eyres, L.

Background: MCT oil is a synthetic oil made from medium chain C8 and C10 fatty acids. It has been traditionally used as a neutral “placebo” in lipid clinical trials. We describe such a trial that raises some concerns.

Aim: To consider the effects of MCT oil, used as a “placebo”, in comparison with an omega-3 polyunsaturated fatty acid-containing supplement, on fatty acid profiles and inflammation in healthy people.

Study Design: Double-blinded, randomised, placebo controlled trial with cross-over, 4 weeks each study arm.

Method: C-reactive protein (CRP) levels were measured in the blood plasma. These levels rise in response to inflammation. Using serum, the fatty acids were measured and analysed by FAME analysis.

Results: Blood samples were collected at each time point, and participants assessed for lipid profiles and CRP. The outcomes of interest were taken from the differences between measures for each phase of the trial. A general linear mixed model was fitted to compare the effect of the MCT before and after the intervention, and in comparison with the omega-3 supplement, with adjustments for BMI, gender, and former smoking status. The fatty acids EPA, DHA and DPA, showed significant decreases while CRP showed a significant increase.

Conclusion: The results revealed unexpectedly adverse effects of the MCT oil supplementation. These comparisons raise questions about MCT oil as a placebo. It was not neutral in its effect on blood lipid biomarkers, or on inflammation. The results have ramifications for claims made about the positive benefits of MCT oils.

### 2.20. Human Breast Milk Complex Lipids: Concentrations, Benefits and the Implications for Paediatric Nutrition

Fong, B.; MacGibbon, A.; Ma, L.; Rowan, A.; McJarrow, P.

Background/Aims: Complex milk lipids include phospholipids, and gangliosides which are found as part of the human milk fat globule membrane. Reported levels of these bioactive components in human milk and human milk substitutes are variable.

Methods: Chinese and Malaysian human milk lactational changes in ganglioside and phospholipid concentrations were analysed using HPLC-MS over 12 months.

Results: The highest total ganglioside and phospholipid concentration in Malaysian and Chinese mother’s milk was at 6 and 12 months lactation respectively. GM_3_ was the most dominant ganglioside class observed in mature milk, while phosphatidyl ethanolamine and sphingomyelin were the two most dominant phospholipid classes observed.

Numerous molecular species were also observed within each ganglioside and phospholipid class as a result of the variety of fatty acids attached to them.

Conclusion: Using modern analytical HPLC-MS techniques, we were able to measure and characterise the human milk ganglioside and phospholipid concentrations. As human breast milk is the gold standard to which infant formulas are formulated, there is awareness that numerous bioactive lipid components are present in breast milk at significant levels, but are at lower concentrations in infant formula. There may be a need to provide these as appropriate building blocks for infant formula to support normal growth and development of the immune system, as well as visual and cognitive performance. Whether this is the case needs to be determined by further research.

### 2.21. A History of Nutrition and War: From Ancient China to Gallipoli and Beyond

Forbes-Ewan, C.

Access to adequate quantities of nutritious food has always been a prerequisite to success in war. In military terms, food can be a ‘force multiplier’.

About 2500 years ago the Chinese general and military philosopher Sun Tzu recommended that invading armies forage on the enemy rather than take food. Half a millennium later, the Romans had discovered that if a general relied on foraging, there was a good chance his army would starve. Roman legionaries commonly carried foods such as ‘hard tack’ biscuits, dried or salted meat and cheese. Combined with foraging, these simple but reasonably nutritious ‘patrol rations’ allowed the Roman Empire to conquer the known world.

There was little progress in food and nutrition for waging war from Roman times until the 18th century, when James Lind—a surgeon in the Royal Navy—showed that citrus juice is antiscorbutic (prevents and cures scurvy). The Royal Navy put this knowledge to good effect: scurvy was virtually absent on British naval vessels after Lind’s discovery. Similarly, but about 150 years later, the Japanese Navy found that reducing the quantity of refined rice in the diet of sailors, and adding meat, fish, flour, milk and vegetables (including beans) led to the elimination of beri beri.

Although the first military application of the principles of nutrition occurred during the Franco-Prussian War of the 1870s—with rationing being based on the estimated protein, carbohydrate and fat requirements of soldiers—the involvement of scientists in the formulation of national food policy in wartime did not occur until World War I. While the UK accepted the need for protective factors in food besides protein, fat, carbohydrate and salts—and that some of these components occurred in fruits and vegetables—Germany underestimated the importance of these factors (which are now known as ‘vitamins’), perhaps influencing the course of the war.

However, there were still problems with getting adequate food to troops during World War I. There is no better illustration of this than the siege of Kut in 1915, when enemy troops surrounded the town, preventing resupply of rations. More than 10,000 British and Indian troops were forced to surrender following five months of increasing levels of malnutrition and outright starvation.

The Gallipoli campaign was also affected by food- and nutrition-related issues. Although food was usually plentiful at Gallipoli, Allied troops (including Anzacs) were fed a diet that was deficient in micronutrients, especially vitamin C. By the end of the campaign, the Anzac troops who survived the fighting had been rendered unfit for military duties as a result of their appalling diet.

The feeding of troops improved dramatically during World War II, with the USA leading the development of a wide range of nutritious combat rations for field feeding.

Developments in military-related nutrition science and food technology continued after World War II, to the extent that troops from developed nations are now supplied foods designed to meet not only their nutritional requirements, but also their taste preferences and cultural needs.

### 2.22. Evaluation of Nutrition Risk in Independent Living Older Adults within the Waitemata and North Shore Community

Fraser, E.; Allen, J.; Gammon, C.S.; Wham, C.A.

Background/Aims: Older people are known to be at a disproportionate risk of malnutrition and have an increased risk of developing health problems as a result of inadequate food and nutrition intake. The aim of this study was to determine the prevalence of nutrition risk amongst independent living older adults >65 years residing in the Waitemata District Health Board catchment, using the Mini Nutritional Assessment-Short Form (MNA-SF) and to identify socio-demographic or health factors associated with nutrition risk.

Methods: Socio-demographic and health data were obtained using a standardised questionnaire completed during a face to face interview. Nutrition risk was assessed using the MNA-SF.

Results: Participants comprised 57 older adults aged 65 to 93 years. Most (93%) had normal nutrition status (MNA-SF score ≥12) whilst 7% were at-risk of malnutrition (MNA-SF score ≤11). The majority of participants with normal nutrition status were NZ European (58%), married (60%) and lived with others (77%), took <5 medications (74%), had fewer (1–3) co-morbidities (70%) and were dentate (42%). Of the 4 participants at risk, all were women, 3 were of Maori and Pacific ethnicity and 3 took ≥5 medications and required support services or daily help.

Conclusion: This study found a low prevalence of nutrition risk in a sample of healthy community-dwelling older adults. Nutrition screening is important to identify those who are vulnerable. Early intervention can help to prevent nutritional and health problems from occurring and to enable older adults to remain active and healthy within the community.

### 2.23. Nutrition and Gut Inflammation—Lessons from Inflammatory Bowel Disease

Gearry, R.

The human gut comprises an essential barrier between the external environment and the body. When compared to other organs, it is unique in its multiple roles and the environmental challenges to which it is exposed. The human microbiome, in contact with the gut mucosa, provides a complex antigenic challenge to the mucosal immune system. However, immune tolerance enables a symbiotic relationship to exist, whereby the gut microbiota provides nutrition to the intestinal mucosa through short chain fatty acids, and the human gut provides a habitat for the microbiota.

However, when immune tolerance is lost, inflammation ensues. Inflammatory bowel disease (IBD) comprises Crohn’s disease (CD) and ulcerative colitis (UC). These chronic diseases have no known cure and manifest as a constellation of symptoms including abdominal pain, diarrhea, rectal bleeding and weight loss. The peak age of onset is 15–40 years with considerable morbidity in young people, affecting education, employment, relationships and quality of life. Treatment comprises immune suppressing medications and surgery, although modification of gut microflora through diet, pro and antibiotics may be helpful.

Recent advances have demonstrated the importance of nutrition in the aetiology of IBD, particularly with interactions between diet and genetic polymorphisms. Complications of IBD also include simple micronutrient deficiencies, which are often under-recognised by clinicians. Less common but devastating is intestinal failure, which may result from recurrent inflammation or complications of repeated gut surgery. These patients require long term total parenteral nutrition (TPN), a costly treatment with significant associated morbidity and mortality or, rarely, intestinal failure.

Dietary manipulation may improve the symptoms of IBD through reducing inflammation in the form of exclusive enteral nutrition (EEN) or through reducing associated functional symptoms with low FODMAP diet. However, patients are confronted with a plethora of advice concerning appropriate diets with few data to support most recommendations. Future research must focus not only on the efficacy of specific diets for IBD, but also the mechanisms behind potential benefits.

Conclusion: Finally, an in depth understanding of perturbations of the gut microbiota in IBD may give insights into the pathogenesis of other illnesses such as irritable bowel syndrome (IBS). While inflammation may only play a small part in the pathogenesis of IBS, it may also enable us to define a healthy gut and develop foods and diets that improve or prevent gut disease.

### 2.24. How Does the Policy Context Affect the Implementation of the National Healthy Start Workforce Project (HSWP)?

Gunn, C.; Congalton, D.; Jaquiery, A.; Barker, M.; Lawrence, W.; Vogel, C.

Background/Aims: The aim of this study is to investigate how policies support implementation of a workforce education and behaviour change programme to improve the diets and lifestyles of pregnant women.

Methods: Local and national policies, resourcing structures and models of service delivery can impact health interventions. The HSWP provides an opportunity to assess how government and organisational policies support delivery of a population-level intervention to help pregnant women adopt healthier diets and lifestyles. Drawing on MRC guidance for evaluating complex interventions, a policy analysis and semi-structured interviews with the maternal and child workforce were completed to explore contextual factors facilitating or hindering intervention implementation.

Results: National and local policy documents relevant to improving the lifestyles of pregnant women were analysed to identify the key Maternal and Child Health priority areas recognised within the policy documents against the WHO Action Plan for NCD prevention policy options for healthy eating and physical activity. Findings from the policy analysis and interviews were used to describe the barriers and facilitators of service delivery for implementing the HSWP intervention.

Conclusion: Rising levels of childhood obesity and the need to address developmental origins of health is increasingly recognised by governments around the world. The HSWP offers a sustainable workforce development solution that could be translated into action in other countries. The key contextual factors enabling or hindering implementation of the intervention provide insight for effective uptake in other communities.

### 2.25. A Literature Review Translates Recent Scientific Research to Support a Maternal and Infant Health Workforce Education Programme

Gunn, C.; Gunn, J.; Jaquiery, A.

Background: The aim of this study is to investigate how a literature review informs Gravida’s Healthy Start Workforce Project (HSWP) education programme on nutrition and physical activity for pregnant women and young children.

Methods: A literature review provided a platform of evidence for the HSWP e-learning programme on the importance for long term health of nutrition and physical activity during pregnancy and early life. Database searches included ScienceDirect, PubMed, Cochrane, DOAJ and Google Scholar with keywords: Pregnancy, nutrition, physical activity, breastfeeding, complementary feeding and DOHaD. Gray literature included WHO and Ministry of Health (NZ) guidelines/reports. Evidence graded highest to lowest included Cochrane reports/meta-analyses/systematic reviews/randomised controlled trials/observational studies.

Results: The research literature demonstrates that during pregnancy, macro/micronutrient intake, weight gain, physical activity and gestational diabetes affect maternal health and the intrauterine environment. The fetal nutritional environment influences gene expression via epigenetic changes and is likely to have intergenerational effects. Breastfeeding and dietary patterns (0–2 years) also may influence infants’ long term health and BMI.

Conclusion: Maternal workforce education must prioritise and reflect current research evidence demonstrating the compelling need for healthy eating and physical activity during pregnancy and early life for long term individual and societal health and economic benefits.

### 2.26. Compliance with Wrist-Worn Accelerometers in 9 to 11 Year Olds: PEDALS

Harrex, H.; Davison, B.; Saeedi, P.; Skeaff, S.; Quigg, R.; Stoner, L.; Black, K.; Meredith-Jones, K.; Skidmore, P.

Background/Aims: One aim of PEDALS (Physical activity, Exercise, Diet And Lifestyle Study) is to determine whether sleep timing is associated with diet and activity in children, independent of total sleep duration. Traditionally accelerometers have been worn at the wrist to measure sleep and the waist to measure physical activity. However, we are interested in measuring both behaviours concurrently and although wrist-worn devices show higher compliance in adults, this has not been shown in school-aged children.

Methods: PEDALS is a cross-sectional study of 400 children (age: 9–11 years) from Dunedin primary schools. Participants were asked to complete lifestyle questionnaires during one school day, and to wear an accelerometer (Actigraph GTX3+) on their non-dominant wrist for eight consecutive days to assess sleep and activity patterns. This sub-study aims to assess compliance to a 24-h wear protocol using a wrist worn-device in school-aged children.

Results: Data collection for PEDALS is ongoing. By July 2015, data was available from 148 participants from seven schools. Preliminary data analysis showed that all participants in the study chose to wear an accelerometer, with 92% of participants providing data for all eight days, and 97% providing data for at least five days.

Conclusion: Initial findings show that uptake of accelerometry measures in this age group is high. This study will be one of the first to determine whether sleep timing influences diet and activity in children, independent of total sleep duration.

### 2.27. The Link between Breast Milk, Infant Faecal and Maternal Faecal Microbiota

Healey, G.; Blatchford, P.; Hedderley, D.; Dinnan, H.; Martell, S.; Herath, T.; Wallace A.; Butts, C.

Background/Aims: To measure the microbiota present in breast milk (BM), infant faeces (IF) and maternal faeces (MF).

Methods: In an observational study, sixty-nine healthy women and their infants donated BM, IF and MF at 6–8 weeks postpartum. Bacterial DNA was extracted from the breast milk and faeces using a MO-BIO PowerSoil DNA isolation kit. An initial PCR was run to amplify the V3-V4 hypervariable region of the 16S rRNA gene. After the PCR products were purified they were sequenced using the Illumina MiSeq Next-Generation sequencing platform. QIIME software was used to analyse the sequencing data, with reference against the Greengenes core reference database.

Results: Strong positive correlations (Spearman’s rank-correlation coefficient of >0.7) were found between *Roseburia* in BM, MF and IF; *Faecalibacterium* in BM, MF and IF; Ruminococcaceae in BM, MF and IF; Lachnospiraceae in BM, MF and IF; *Lachnospira* in BM and IF; *Veillonella* in BM and MF and *Ruminococcus* in BM and MF.

Conclusion: There were significant overlaps in the bacterial taxa present in the breast milk, infant faeces and maternal faeces. This observational research suggests that, through mechanisms not yet fully understood (i.e., entero-mammary pathway), maternal gut-associated microbiota may be shared with the infant via breastfeeding. This may be a key step in the colonisation of the infant gut by commensal gut-associated microbiota.

### 2.28. Dietitians’ and Nutritionists’ Utilisation of Counselling Approaches to Facilitate Eating Behaviour Change: Pilot Study

Hintz, E.; Weber, J.L.; Coad, J.; Thomson, J.A.

Background: The need for counselling skills has long been acknowledged in nutrition and dietetics. Traditional approaches have been based on didactic education and advice giving. However, in the past 30 years there has been a documented shift to integrate Cognitive-Behavioural Therapy (CBT) and Motivational Interviewing (MI) techniques.

Aims: To investigate New Zealand dietitians’ and nutritionists’ perceived adequacy of training and confidence in using behaviour change techniques.

Methods: Members of Dietitians NZ and Clinical Nutrition Association were invited to complete a questionnaire about their use of behaviour change techniques. Twenty three finished questionnaires were returned.

Results: Most (91%) participants were dietitians. Around half (48%–57%) believed their training in person-centred counselling was adequate/more than adequate and most (61%) indicated adequate training in MI; but not all facets. Most (83%) described their main approach to dietary treatment as ‘person-centred’ and most (70%–87%) felt confident/extremely confident using person-centred and MI with clients. Around half (44%–61%) were confident/extremely confident using most facets of CBT and mindfulness-based eating. Most (87%) felt their clients had moderate to very good success rate over the short-term, but less than half (43%) felt that a dietitian alone was the best trained professional to manage eating behaviour change. Confidence using techniques may be due to most (87%) participants undertaking additional training in behaviour change techniques.

Conclusion: Most participants felt confident using a variety of counselling approaches, particularly person-centred and MI, but most had attended ongoing training on behaviour change techniques.

### 2.29. The Effect of Vitamin D on Chronic Plaque Psoriasis: A Randomised, Double-Blind, Placebo-Controlled Supplementation Trial

Ingram, M.A.; Jones, B.; Stonehouse, W.; Jarrett, P.; Scragg, R.; von Hurst, P.

Background/Aim: Vitamin D has anti-proliferative, pro-differentiative and immunomodulating effects. We aimed to determine whether raising serum 25(OH)D through vitamin D_3_ supplementation improves psoriasis.

Methods: In a randomised (2:1), double-blind, placebo-controlled trial, 101 participants >18 years with plaque psoriasis took 200,000 International Units (IU) of cholecalciferol at baseline then 100,000 IU/month for 11 months (*n* = 67), or placebo (*n* = 34). Psoriasis Area and Severity Index (PASI) and serum 25(OH)D concentration were assessed at baseline, 3 months, 6 months, 9 months and 12 months. Primary outcomes were a) difference in PASI between groups over time, and b) the relationship between PASI and 25(OH)D over time, assessed by linear mixed models adjusted for confounding/individual factors.

Results: There was a significant inverse relationship between 25(OH)D and PASI. Elevating serum 25(OH)D by increments from 25–125 nmol/L was associated with mild decreases in PASI (estimated range of decrease 0–2.6; *p* = 0.002). PASI did not differ by group (*p* = 0.62, group by time *p* = 0.54), and an improvement in PASI of 50% or higher was achieved by 11.9% of treatment and 11.8% of placebo. However, mean 25(OH)D significantly increased from baseline at 3 months for treatment (*b* = 33 [95% CI 28–38] nmol/L, *p* < 0.001) and 6 months for placebo (*b* = 24 [95% CI 17–30] nmol/L, *p* < 0.001), possibly confounding these results.

Conclusion: At a population level, elevating serum 25(OH)D is associated with improved psoriasis. Estimated improvements were mild at 25(OH)D concentrations in this study and may not be clinically significant; higher concentrations could have greater benefit.

### 2.30. Does Sweet Taste Perception Explain Habitual Sweet Food Liking and Choice?

Jayasinghe, S.N.; Kruger, R.; Walsh, D.C.I.; Rivers, S.; Breier, B.H.

Background/Aims: Sensory attributes such as smell, taste and texture influence dietary behaviour. Given that sweet taste has a powerful hedonic appeal; preference for sweet tasting foods may contribute to excessive consumption. The aim of the study was to investigate whether sensitivity to and preference for sweet taste influences sweet food liking and choice.

Methods: This cross-sectional study recruited 45 women aged 20–40 years. Taste analysis included glucose recognition threshold and rating sweet taste intensity and hedonic liking of 125 mM, 250 mM, 500 mM and 1000 mM glucose samples. Three questionnaires assessing eating behaviour and sweet food liking and choice were completed.

Results: Recognition threshold data indicate that some individuals are more sensitive to sweet taste than others. A negative relationship was observed between sweet taste intensity and hedonic liking of 1000 mM glucose (*r* = −0.78, *p* < 0.001). Furthermore, sweet taste intensity of 1000 mM glucose was negatively correlated with fruit juice (*r* = −0.44, *p* = 0.002) and fruit drink (*r* = −0.47, *p* = 0.001) liking. Fruit juice liking correlated positively with the hedonic liking of the 1000 mM glucose solution (*r* = 0.35, *p* = 0.02). Participants preferring savoury foods as snacks, rated 1000 mM glucose as more intense (*t*(43) = −2.26, *p* = 0.03).

Conclusion: Liking of sweet beverages is associated with reduced perceived intensity and higher hedonic liking of sweet taste. Furthermore, the sweet intensity perception appears to influence the choice of snack food. Our data suggest that intensity perception and hedonic preference of sweet taste play important roles in habitual sweet food liking and choice.

### 2.31. How Healthcare Professionals and Breastfeeding Women in New Zealand Use Food and Herbal Galactogogues: A Pilot Study

Jia, L.; Weber, J.; Brough, L.

Background: Perceived inadequate milk production is a main cause of early cessation of breastfeeding. Many herbs and foods (galactogogues) are used worldwide to enhance breast milk production based on anecdotal evidence around efficacy and safety. There is no scientific literature about the use of galactogogues in New Zealand (NZ).

Objectives: To describe both healthcare professionals’ and breastfeeding women’s attitude to and experiences with galactogogues in NZ.

Methods: Healthcare professionals (*n* = 8) involved in postnatal care and breastfeeding women (*n* = 7) who had previous concerns with milk supply participated in a tailored semi-structured interview. Content analysis was adapted for coding and analysing transcripts.

Results: Foods used as galactagogues included oats, dairy products, and increasing protein and “healthy fat” and supplementing vitamins were reported. A range of herbs were used, with fenugreek most commonly mentioned. Women with long-term problems usually were prescribed domperidone, a pharmaceutical galactogogue. Healthcare professionals generally tried to eliminate perceived problems and apply breastfeeding techniques including frequent feeding, breast compression and expressing after feeding before introducing galactogogues. Women sourced information from other breastfeeding women or the Internet, and sometimes tried galactogogues before contacting professionals for help. Most respondents raised concerns about safety, although some considered ‘natural’ safer whereas others were more confident about pharmaceutical galactogogues.

Conclusion: Galactogogues are being used in NZ, even though there is limited scientific evidence of safety or efficacy. More research is required to gain a fuller understanding of the use of galatogogues in NZ.

### 2.32. Fish and Shellfish Intake Prior to and during Pregnancy among Qualifying Women in New Zealand

Jin, Y.; Coad, J.; Weber, J.; Gris, F.; Brough, L.

Background: Fish consumption is beneficial for foetal neurodevelopment due to the presence of omega-3 fatty acids and other micronutrients. The Ministry of Health provides recommendations regarding restricting fish/shellfish consumption in pregnancy due to concerns regarding food safety and mercury contamination.

Aims: To determine fish/shellfish intake in women before and during pregnancy.

Methods: Pregnant (66) and breastfeeding (87) women were recruited throughout New Zealand. Fish/shellfish intake was determined by a researcher led, food frequency questionnaire. Pregnant women recalled fish/shellfish consumption prior to pregnancy, while breastfeeding women recalled intake during pregnancy.

Results: Seventy percent were Caucasians and 85% had university degrees. All women ate fish/shellfish pre-pregnancy and 91% during pregnancy. Few women reported having fish/shellfish less than once weekly before pregnancy (9%) increasing to 24% during pregnancy (*p* = 0.018). Around half (46%) of women ate fresh fish more than once a week before pregnancy, decreasing to 30% during pregnancy (*p* = 0.041). Conversely, 39% of women ate canned fish more than once weekly before pregnancy, non-significantly rising to 47% during pregnancy. Of the participants, 37%–45% consumed shellfish.

Conclusion: The majority of women consumed fish while less than half of women ate shellfish. Most women tended to eat fish/shellfish more than once a week. Fish intake decreased in pregnancy compared to before pregnancy. Further research is required to understand the reasons for reduced fish/shellfish intake in pregnancy.

### 2.33. The Fortification of Bread with Iodised Salt Eradicates Iodine Deficiency in New Zealand Schoolchildren

Jones, E.; Davies, B.; Hawkins, R.; Meiklejohn, E.; McLean, R.; Skeaff, S.

Background/Aim: Iodine deficiency re-emerged in New Zealand (NZ) in the 1990s, prompting the mandatory fortification of bread with iodised salt from 2009. This study aimed to determine if the fortification of bread has improved the iodine status of NZ schoolchildren.

Methods: This was a cross-sectional survey of 8–10 years old children living in Auckland and Christchurch conducted from March–May 2015. Children provided a spot urine sample for the determination of urinary iodine concentration (UIC) and completed a questionnaire ascertaining socio-demographic characteristics that also included an iodine-specific food frequency questionnaire (FFQ). The FFQ was used to estimate iodine intake from main food sources including bread and iodised salt.

Results: The median UIC for all children (*n* = 415) was 116 μg/L (females: 107 μg/L; males: 131 μg/L), indicative of adequate iodine status according to WHO (i.e., 100 to 199 μg/L). There were signficant differences in UIC by ethnicity (*p* < 0.001) and sex (*p* = 0.006) but not iodised salt use (*p* = 0.890). The mean estimated iodine intake including iodised salt was 101 μg/day (females: 101 μg/day; males: 102 μg/day), with 23% of the children having an iodine intake below the EAR. Bread contributed 51% of total iodine intake in the food-only model, providing a mean intake of 35 μg/day.

Conclusion: These results are comparable to a similar study conducted in 2011 of Dunedin and Wellington children. Together they provide convincing evidence that iodine deficiency has been eradicated in NZ schoolchildren since the mandatory fortification of bread with iodised salt.

### 2.34. New Zealand Muriel Bell Lecture: New Paradigms for Old Bones

Kruger, M.

The skeleton is a metabolically active organ that is continuously resorbed and rebuilt. With ageing, the balance shifts to increased resorption, leading to osteoporosis due to a reduction in bone mineral density (BMD) and disruption of bone microarchitecture. Bone metabolism is influenced by hormones such as oestrogen, thus post-menopausal women worldwide have a heightened risk of developing osteoporosis. Lifestyle factors also affect risk, with diet and exercise being the most important modifiable factors. Dietary calcium, vitamin D, fruit, vegetable and long chain polyunsaturated fatty acids (PUFAs) intake all correlate with bone health.

Osteoporosis is diagnosed using several methods. The measurement of BMD by dual energy X-ray absorptiometry (DXA) is the gold standard. Portable ultrasound heel scanners are practical for assessing bone health status in large population studies. Calcium and vitamin D serum levels provide valuable information about nutritional status. Changes in BMD occur very slowly, interventions can be monitored via blood levels of bone-turnover markers including c-telopeptide of type I collagen (CTX), osteocalcin, and receptor activator of nuclear factor kappa-B ligand (RANKL), with parathyroid hormone (PTH) as indicators of calcium balance.

Collaborative work with New Zealand and international research groups has focused on using foods and food components to intervene in the bone resorption process. Initial studies were carried out in vitro to identify cellular and molecular mechanisms as well as optimal dosages. Animal studies provided in vivo evidence, while human studies demonstrated efficacy at both individual and population levels.

In vitro studies demonstrated that the PUFA docosahexaenoic acid (DHA) from fish oil, β-carotene, and the oestrogen-like soy isoflavone daidzein reduced osteoclast formation, bone resorption, and osteoclast differentiation markers. These findings were extended to animal models. In ovariectomised (OVX) rats, which mimic the post-menopausal woman, dietary supplementation with DHA and other PUFAs significantly increased BMD and decreased serum PTH. Feeding β-carotene-rich kiwifruit and soy isoflavones significantly reduced CTX and RANKL serum levels and improved BMD in OVX rats and mice. Human studies similarly demonstrated the benefits of dietary supplementation. In healthy postmenopausal women, increasing vegetable and fruit intake reduced calcium excretion in the urine, kiwifruit consumption improved bone markers and fortified milk significantly reduced bone resorption.

However, human studies also revealed significant differences in genetic phenotypes, debunking long-held beliefs and leading to new paradigms in study design. A comparison of women in several Asian countries identified significant differences between the rate of decline in bone health in women in Vietnam compared to women in Indonesia and the Philippines. A study of black women in Africa demonstrated that higher proportions of body fat, once believed to protect against osteoporosis, did not correlate with bone health. Rural African women had higher 25 (OH) D levels but also higher bone turnover compared to urban women. Isoflavone interventions in Asian postmenopausal women have produced inconsistent bone health benefits, due in part to population heterogeneity in enteric bacterial metabolism of daidzein. Ongoing studies are now being specifically designed to assess genotype differences between Western, Malay, Chinese, African, and other phenotypes.

### 2.35. Exploring Eating Behaviour Using the Three Factor Eating Questionnaire (TFEQ) in Overweight and Obese Women

Kruger, R.; de Bray, J.G.; Beck, K.L.; Conlon, C.A; Stonehouse, W.

Background: Obesity is a leading cause of morbidity and mortality, yet it is preventable. Interventions which fail to address behavioural contributors to weight gain are unlikely to be successful. This study aimed to investigate the relationship between body mass index (BMI), body fat percentage (BF%), and obesity related eating behaviours.

Methods: Women (*n* = 116), aged between 20 and 45 years, were recruited. Quetelet’s BMI was calculated (kg/m^2^) from height and weight measurements, and BF% was measured using air displacement plethysmography (BodPod). Women completed the validated Three Factor Eating Questionnaire (TFEQ) to assess their eating behaviours. The three main eating factor scores (Restraint, Disinhibition and Hunger) and their sub-categories were calculated. The TFEQ data were analysed for associations with BMI and BF%, as well as categorisations based on high BMI (≥25 and ≥30 kg/m^2^) and high BF% (≥30%).

Results: The women had a median (IQR) age of 34 (27, 40) years, BMI of 23 (21, 25) kg/m^2^, and mean ± SD BF% (30.5% ± 8%). Disinhibition was positively correlated with both BMI (*p* < 0.001) and BF% (*p* < 0.001). Emotional Disinhibition was the only TFEQ sub-category that differed significantly between women with high versus normal BMI and BF%. No significant correlations were found between Restraint or Hunger and BMI or BF%.

Conclusion: Disinhibition seems likely to be a key contributor to both higher BMI and BF%. Intervention strategies which educate overweight or obese women on how to counteract Disinhibition should be a key target area for prevention of further weight/fat gain.

### 2.36. Maternal Overweight Is Associated with Infant Feeding Patterns and Growth

Lagström, H.; Makela, J.

Aims: To evaluate how maternal overweight impacts exclusive breastfeeding (BF), total duration of BF and the age of introduction of complementary foods (CF) and whether these have effect on children’s growth from 0 to 2 years.

Methods: From 1797 families participating to the Finnish STEPS Study, 848 children had data on breastfeeding and anthropometric data from 13 months and 2 years of age. Data on BF and CF were collected by self-administered follow-up diary. Information regarding maternal weight, height, pregnancy and delivery were received from maternity clinics and the National Longitudinal Census Files. Children’s weight and length/height were recorded during the study visits at 13 months and 2 years.

Results: Overweight women breastfed exclusively (2.2 vs. 2.8 mo, *p* < 0.001) and totally (7.4 vs. 9.0 mo, *p* < 0.001) shorter time and introduced CF earlier (4.1 vs. 4.3 mo, *p* = 0.02) than normal weight women. Children of overweight women were heavier and had higher BMI at 2 years than children of normal weight women. At 2 years of age 30% of boys and 17% of girls were overweight or obese. Children’s obesity risk was not increased by maternal overweight (OR 1.04, *p* = 0.12). Longer duration of full BF (OR 0.86, *p* = 0.04) and partial BF (OR 0.91, *p* = 0.02) and later introduction of CF (OR 0.69, *p* = 0.03) were protective against obesity.

Conclusion: Our study suggests that women who were overweight or obese before pregnancy breastfed for shorter time and introduced CF earlier than normal weight women, which may further impact children’s growth.

### 2.37. The Effects of the Supplement ‘Lester’s Oil’ on Fatty Acid Concentrations and an Inflammation Biomarker in Healthy People

Laing, B.; Ellett, S.; Marlow, G.; Jesuthasan, A.; Agnew, M.; Ferguson, L.

Introduction: Diet is significant in the disease susceptibility of individuals. Omega-3s are associated with immune regulatory functions. Omega-3 supplementation has shown reduced inflammation especially in people with inflammatory disorders. To achieve this, the preparation has to be bioavailable.

Background/Aim: To investigate the effects of an anti-ageing supplement, Lester’s Oil (About Health^®^ Supplements Ltd, Auckland, NZ) on fatty acid profiles and inflammation (as measured by CRP) in healthy people.

Study Design: Double blinded, randomised, placebo controlled with cross-over.

Methods: Plasma C reactive protein (CRP) levels, which rise in response to inflammation, were measured. Serum fatty acids were measured and analysed by FAME analysis. The outcomes of interest were taken from the differences between measures for each phase of the trial. A general linear mixed model was fitted to compare the effect of the supplement with a medium chain triglyceride oil with adjustments for differences before and after the trial for BMI, gender, and former smoking status.

Results: In response to supplementation the fatty acids EPA, DHA and DPA showed significant increases (*p* < 0.001; *p* < 0.001; *p* < 0.001); the fatty acids Oleic acid, Palmitoleic and Myristoleic showed significant decreases (*p* = 0.003, *p* = 0.016, *p* = 0.022); and CRP showed a significant decrease (*p* = 0.011).

Conclusion: These results show the supplement was effective in improving key fatty acids (EPA, DHA, DPA) and decreasing inflammation in healthy people.

### 2.38. US Military Nutrition: Historical Approaches and Contemporary Perspective

McClung, J.

Nutrition is essential for maintaining the optimal health and performance of Warfighters. Failure to provide energy and nutrients at required levels results in measurable declines in cognitive and physical performance and, if chronic, may be associated with distinct nutritional deficiency disorders. The objective of this presentation is to detail the history of military nutrition and ration development as it applies to US Warfighters and to provide a contemporary prospective regarding current and future military nutrition research and practices.

Initial recognition of the importance of nutrition to Warfighter health in the US came in 1775 when the Continental Congress stipulated that enlisted members of the Continental Army should receive meat, milk, bread, and vegetables. Until World War I, personnel were provided with the garrison ration, a ration that was served to Warfighters in all circumstances, to include combat and garrison dining environments. During World War I, the first special purpose rations were developed and utilized, to include the reserve ration, the trench ration, and the emergency ration. The period prior to and during World War II was marked by the emergence of a series of field rations, as well as recognition that rations were required for the mountain, jungle, and desert environments. Currently, in field conditions, Warfighters are provided with the Meal, Ready-to-Eat (MRE), which has been designed to meet the nutritional requirements of Warfighters and includes over 20 menus and 150 individual components.

Contemporary studies in the area of US military nutrition research are focused on the development of ration and garrison paradigms that meet Warfighter requirements specific to operational demands. Examples include fortified snack items that can be provided before, during, and after operational missions for the optimization of physical and cognitive performance, resilience, and recovery. Other contemporary topics include meeting the nutritional needs of female Warfighters, to include preventing declines in iron status that may occur during physical training and optimizing vitamin D and calcium status in an effort to sustain bone health. Future approaches are likely to incorporate the nutritional requirements of individual Warfighters, including the contribution of genetic factors such as single nucleotide polymorphisms.

The views expressed in this abstract are those of the authors and do not reflect the official policy of the Department of Army, Department of Defense, or the U.S. Government.

### 2.39. Estimates of Sodium and Potassium Intake in a New Zealand Adult Population Sample—Have We Got the Balance Right?

McLean, R.M.; Edmonds, J.C.; Williams, S.M.; Mann, J.I.; Skeaff, S.A.

Background/Aims: To assess sodium and potassium intake in New Zealand adults using 24 h urine samples.

Methods: A random sample of New Zealand adults aged 18 to 64 years was selected from the electoral roll in two cities: Dunedin and Wellington. Participants completed a questionnaire with sociodemograhic information, had height, weight and blood pressure measured, and provided a 24 h urine sample, which was analysed for sodium and potassium content.

Results: Mean sodium:potassium ratio was 1.32 (95% CI 1.26, 1.39); 1.39 for men and 1.26 for women. Mean 24 h sodium excretion was 3386 mg/day (95% CI 3221, 3551): 3865 mg/day for men and for 2934 mg/day women. Mean 24 h potassium excretion was 2738 mg/day (95% CI 2623, 2855): 3031 mg/day for men and 2436 mg/day for women. Sodium intake was higher among younger people, men, those with a higher BMI and higher potassium excretion. Potassium excretion was higher among older people, men and those with a higher sodium excretion.

Conclusion: New Zealand adults have high sodium intakes and low potassium intakes compared to recommended levels. Dietary sodium intakes have remained relatively stable since the late 1970s. A comprehensive public health programme to reduce dietary sodium intake and increase intake of fruit and vegetables would help reduce population blood pressure and incidence of cardiovascular disease.

### 2.40. Eating Habits and Nutrition Attitudes among Pregnant Chinese Women in New Zealand

Ma, J.; Coad, J.; Brough, L.; Weber, J.L.

Background: The number of immigrants from China has increased significantly over recent years. Pregnant Chinese women in New Zealand may face a number of barriers in following nutrition recommendations.

Aims: To assess nutrition attitudes and practices of pregnant Chinese immigrants in New Zealand.

Methods: Eighty four pregnant Chinese women living in New Zealand were recruited via Chinese community websites and organisations. Information on eating habits, attitudes towards NZ and Traditional Chinese Medicine (TCM) nutrition recommendations, and acculturation was collected using an online questionnaire.

Results: The majority of participants had a positive attitude towards NZ nutrition recommendations for pregnant women, but reported consuming fewer than the recommended serves from food groups. The majority of women reported taking folic acid supplements at least 5 days/week (77%), but less than half took iodine supplements (40%) or iodine rich foods (17%) as often. Most had positive attitudes towards TCM recommended dietary practices for general health, e.g., adjusting diet to body composition (70%), but most (>70%) seldom consumed foods with TCM features specifically recommended for pregnancy. More acculturated women had more positive attitudes towards both NZ and TCM nutrition recommendations and were more likely to consume recommended serves from foods groups.

Conclusion: Attention needs to be paid to pregnant recent Chinese migrants to help them meet recommended serves from food groups and ensure they understand the need for iodine and iodine supplements in New Zealand. Health professionals should be familiar with general TCM dietary recommendations.

### 2.41. Military Nutrition—Turning Research into Practice: The New Zealand Defence Force Perspective

Martin, N.

The saying “an army marches on its stomach” was first recorded in English in the early 20th Century. Although modern warfare is no longer as heavily dependent on the foot soldier *per se*, this saying remains relevant today with respect to fuelling our ‘Combat Athletes’. Alongside promoting the maintenance of optimal health, good nutrition enhances the operational readiness of military personnel by improving their physical and cognitive performance. This is achieved through its effects on energy stores, hydration status, body composition, recovery, injury prevention, immune function and sustaining morale. We have come a long way from the monotonous, energy- and nutrient-deficient rations New Zealand soldiers received during World War I, where many of their meals consisted of canned meat (bully beef), jam, hard biscuits and a hot brew. More than ever, mission success requires today’s Combat Athlete to optimise a multi-factorial skill set, including endurance, strength, speed, agility, accuracy and decision-making.

This presentation will focus on turning military nutrition research into practice within the New Zealand Defence Force. It will primarily look at projects within the NZ Army to support Combat Athletes—past, present and future. Past—Projects focused on delivering nutrition education to both soldiers and commanders; modifying the food environment for garrison, exercises and deployments. Present—Ongoing refinement of the food environment; modification of operational rations to meet current training and deployment needs; an investigation into the iron and vitamin D status of female recruits. Future—An increased focus on the multi-disciplinary approach to delivering Performance Health(care) to Combat Athletes; optimising resilience, both physical and mental, will be a key driver.

### 2.42. Health and Nutritional Literacy of New Zealand Nursing Students

Mearns, G.; Chepulis, L.M.

Background: Published literature suggests that nurses in New Zealand are more overweight than the general population. A lack of nutrition knowledge, social eating patterns and shift work are proposed to contribute to this. This study aimed to assess nutrition knowledge of undergraduate nursing students and to measure associations with demographics, lifestyle factors, health status and dietary patterns.

Methods: An observational study with a convenience sample of 103 first-year undergraduate nursing students recruited from two tertiary education institutions in the North Island, New Zealand. Data collection included anthropometry, blood lipids, glucose and HbA1c. Nutrition knowledge was tested using a 22-question nutritional literacy survey.

Results: No correlation was observed between anthropometry measures and total literacy score. Mean BMI value was 26.7 ± 6.3 (range 16.5–44.0) and approximately 60% of participants were either overweight or obese. The mean total correct nutritional literacy score was 56.7% ± 13.2%. Participants scored higher in food label and health-food claim questions (>70% correct) and lower in general nutritional knowledge. The majority of participants were within normal limits on blood test results, although 5% had elevated HbA1c and one third had an elevated total cholesterol.

Conclusion: Prevalence for obesity and overweight amongst nursing students exceeds that of the general female population and may explain the high overweight and obesity prevalence for registered nurses. Findings link risk for elevated BMI among first year nursing students with demographic and lifestyle factors rather than nutritional knowledge.

### 2.43. Comparison of Dietary Patterns of Adults in Brazil and New Zealand

Mendes Borges, T.; Mason, S.

Background/Aims: Comparison of dietary patterns between populations can help to explore the relationships between food habits, factors that affect them, nutritional related diseases and future food security. This study aimed to compare the food intake of adults in Brazil and New Zealand.

Methods: Data was obtained primarily from the 2008–2009 Brazilian Household Budget Survey, individual food intake and the 2008–2009 NZ Adult Nutrition Survey. Food and beverages were combined into 21 food categories and the percent of total energy contributed by each food group was calculated and compared between countries.

Results: The contribution of different food categories to energy intake differed between Brazilian and New Zealand adults. The top 5 categories contributing to energy intake for Brazilians were rice pasta and grains; non-alcoholic beverages; beef; legumes and breads while for New Zealanders, they were legumes; sweets and confectionary; breads; rice pasta and grains and non-alcoholic beverages. From an energy perspective, Brazilians ate three times more beef and twice the rice, pasta and grains but only one quarter of the eggs and one seventh of the sweets and confectionaries than NZ adults ate. Although New Zealand adults drink more milk than Brazilians, the contribution of other dairy products to calorie intake is similar in both countries.

Conclusion: Different dietary patterns are seen in New Zealand and Brazil. Many factors including income and climate affect this. The comparison suggests the growing levels of obesity in these countries may have different dietary causes and require different strategies to limit the increase.

### 2.44. The Impact of Age on the Inflammatory, Endotoxaemic, and Oxidative Stress Responses to a High Fat Meal

Milan, A.M.; Pundir, S.; Pileggi, C.A.; Markworth, J.F.; Roy, N.C.; Cameron-Smith, D.

Background/Aims: Postprandial inflammation, endotoxaemia, and oxidative stress are determinants of cardiovascular and metabolic disease risk and are amplified after high fat meals. We aimed to examine determinants of postprandial inflammation and endotoxaemia in older and younger adults following a high fat mixed meal.

Methods: In a randomised cross-over trial, healthy participants aged 20–25 and 60–75 years (*n* = 15/group) consumed separately a high fat and a low fat breakfast. Plasma taken at baseline and post-meal for 5 h was analysed for circulating endotoxin, cytokines (monocyte chemotactic protein (MCP)-1, IL-1β, IL-6, and tumour necrosis factor (TNF)-α), lipopolysaccharide binding protein (LBP), and expression of inflammatory genes in peripheral blood mononuclear cells (PBMC).

Results: Older subjects had 16% lower baseline PBMC expression of glutathione peroxidase (GPX)-1 (*p* < 0.05), but 2.7-fold greater baseline expression of insulin-like growth factor-binding protein (IGFBP)-3 (*p* < 0.001) and 11% greater circulating MCP-1 compared to younger subjects (*p* < 0.05). After either meal, there were no age differences in plasma or chylomicron endotoxin or plasma LBP concentrations, nor in inflammatory cytokine gene and protein expression (MCP-1, IL-1β, and TNF-α). Unlike younger participants, the older group had a 35% decrease in SOD-2 expression after the meals (*p* < 0.001).

Conclusion: After a high fat meal, older adults had no increased inflammatory or endotoxin response, but an altered oxidative stress response. Healthy older adults, without apparent metabolic dysfunction, have a comparable postprandial inflammatory and endotoxaemia response to younger adults.

### 2.45. Dietary Minerals, Blood Pressure, and Heart Disease—Separating Fact from Fiction

Moore, L.

High blood pressure is a major modifiable risk factor for cardiovascular and cerebrovascular diseases. Non-pharmacologic approaches to the prevention and management of CVD typically include weight loss, stress reduction, smoking cessation, exercise, and diet modification. Sodium restriction has been the primary dietary strategy for blood pressure management for many years. While average sodium consumption is approximately 3.5 g/day across many populations, current U.S., Australia, and New Zealand Dietary Guidelines recommend limiting sodium intake to 2.3 g/day for adults and most adolescents. U.S. Guidelines and some others suggests that some segments of the population, including blacks of any age, everyone over age of 50, and anyone with high blood pressure, diabetes or chronic kidney disease (~50% of the US population), should limit sodium intake to as low as 1.5 g/day. This talk will address a number of provocative questions about dietary sodium. For example, is current salt intake excessive for most individuals and would a lower level of intake generally be better? Would most people be healthier if they consumed less salt, or might salt restriction have unintended consequences on health outcomes such as lipid levels or insulin resistance? Is it even feasible to reduce sodium levels in the general population? Evidence for the effects of dietary sodium and sodium restriction on blood pressure and secondary vascular outcomes will be reviewed. The role of salt sensitivity and its interaction with patterns of dietary potassium intake have also emerged as important factors in dietary advice for the prevention of high blood pressure and related cardiovascular outcomes. In general, the roles of several dietary minerals on cardiometabolic risk seem to have been underestimated. For example, with its ability to affect vascular tone, potassium may play a much more important role in blood pressure regulation and cardiac functioning than has been previously appreciated. Calcium also has a controversial history. For many years, it was believed to be linked with blood pressure reduction but clinical trials have yielded results that are less than convincing. In the original DASH clinical trials, the calcium content of dairy was a major reason for its inclusion in the combined dietary intervention arm of the study. More recent evidence suggests that other factors in dairy may be responsible these beneficial effects instead. Finally, magnesium is a dietary mineral that may have significant impact on vascular health through the promotion of endothelium-dependent vasodilation and maintenance of normal cardiac electrophysiology. The direct effects of magnesium on blood pressure and other secondary cardiovascular outcomes will be reviewed. Dietary minerals are consumed as a part of an overall nutrient package. As a result, it is important that we look at those nutrient packages in an effort to understand the total effects of dietary sodium, potassium, calcium and magnesium on long-term health outcomes. Funding source: National Institute of Diabetes and Digestive and Kidney Diseases (NIDDK), the National Dairy Council in the U.S., the Dairy Council of California, USDA and Dairy Australia.

### 2.46. 25-Hydroxyvitamin D Levels Are Lower in New Zealand Residents with Active Inflammatory Bowel Disease

Morton, H.; Coad, J.; Pedley, K.C.

Background/Aims: Individuals with IBD (Crohn’s Disease (CD) and Ulcerative Colitis (UC)) may be at risk of low vitamin D levels due to reduced absorption, use of sun-sensitising medications, and increased requirements. Low vitamin D levels in IBD have been associated with greater disease activity and longer disease duration.

Methods: 186 men and women with IBD, and 126 healthy controls, provided a blood spot sample to measure serum 25-hydroxyvitamin D level. 198 of these participants completed a retrospective questionnaire containing demographic data, sunlight exposure habits, vitamin D supplementation, and disease activity. Difference between groups was tested using ANOVA; frequency of vitamin D deficiency and adequacy was tested using Chi-Square.

Results: No differences were observed between vitamin D level and disease, latitude, or sun exposure hours. Lower vitamin D was significantly associated with recent disease activity in patients with CD (66.0 ± 3.4; *p* = 0.011), but not UC, and in IBD after controlling for supplementation (57.8 ± 3.1; *p* = 0.018). Higher vitamin D was significantly associated with supplement use (82.3 ± 3.9; *p* < 0.01) and recent increased sun exposure (73.6 ± 2.5; *p* < 0.01).

Conclusion: Vitamin D Supplementation should be considered both during and in the 12 months following flare up of disease activity in IBD.

### 2.47. Maternal Nutrition and the Early Life Origins of Obesity

Muhlhausler, B.

A world-wide series of epidemiological and experimental animal studies have provided evidence that the pathway to obesity can begin very early in life, and that environmental exposures before birth and/or in early infancy play a key role in defining an individual’s susceptibility to obesity through the life course. The nutritional environment an individual experiences during these periods plays a particularly important role in the early life origins of obesity and poor metabolic health. Initial studies in this area focused on the consequences of an inappropriately low or inappropriately high nutrient supply, both of which are associated with an increased risk of obesity in postnatal life and leading to the concept of a U-shaped relationship between early life nutrient supply and later metabolic health. More recently, it has become clear that not only global over or undernutrition, but also excesses and deficiencies of key dietary components, including specific fatty acids, protein and/or key micronutrients, also plays a critical role in the early life origins of obesity and metabolic disease. This has resulted in growing interest in the potential for nutritional interventions applied during pregnancy or early infancy to improve the future metabolic health of children. This presentation will provide a background on the role of nutrition in the early life origins of obesity and our current understanding of the underlying mechanisms, with a particular focus on studies which have set out to test the potential for nutritional interventions in pregnancy or infancy to improve metabolic health outcomes in the child.

### 2.48. Multilevel Multicomponent Intervention Strategies for Childhood Obesity Prevention

Novotny, R.

Childhood obesity is an important determinant of adult obesity. Obesity is associated with social and health problems. Intervention to decrease and prevent further increase in the prevalence of childhood obesity may include child, family, household, community, environment, and policy components. Including multiple components or levels of influence increases the likelihood of impact of programs that aim to decrease prevalence of childhood obesity. The Children’s Healthy Living Program for Remote Underserved Minority Populations of the Pacific (CHL) is a multilevel multicomponent intervention program in five jurisdictions (Hawaii, Guam, Alaska, American Samoa and the Commonwealth of the Northern Mariana Islands) of the United States affiliated Pacific. CHL was designed through combining scientific evidence and community input, implemented by supporting community role models and programs that were engaged in aspects of the identified intervention components, facilitating coalition building, and is being evaluated in a multilevel analytic model and by process evaluation. CHL will be described as an example of developing and implementing multilevel multicomponent childhood obesity intervention programs that aim to make comprehensive systems change. Emphasis will be on strategies and approaches to childhood obesity prevention intervention that can be adapted for diverse populations. Funding source: United States Department of Agriculture, National Institute of Food and Agriculture, Grant 2011-68001-30335.

### 2.49. Optimal Nutrition for Healthy Ageing: More or Less?

Nowson, C.

Life expectancy of both genders is increasing and the proportion of older people continues to rise, with increasing numbers of older people living in the community with a range of chronic diseases that impact on quality of life. We know that adoption of a healthy diet early in life can reduce the risk of, or delay the onset of a range of chronic disease, however it appears that the dietary recommendations that are effective in reducing disease risk in early and mid-life may not be are appropriate for later life. There is considerable evidence indicating that those >65 years require significantly more nutrients than mid-life to maintain functional status and optimal quality of life such as calcium and vitamin D. Muscle strength, which is related to muscle mass, is the key determinant of maintaining activities of daily living. The maintenance of muscle strength requires a combination of resistance exercise and increased amounts of dietary protein. Although there has been a massive increase in the number of older people who are classified as overweight and obese, the evidence to date does not support the implementation of weight loss programs in older people, as it appears that carrying some additional weight over the age of 65 years reduces mortality. As the number of “very old” (those >85 years) increases, where weight loss is common, there is an increasing need to implement widespread nutrition screening in both community and residential care settings. This initiative needs to be supported by education programs, and the development suitable meals and foods that reduce the risk of malnutrition and optimise energy and nutrient intake to maintain quality of life.

### 2.50. Objectively Measured Physical Activity Levels of Obese and Non-Obese Pre-Menopausal New Zealand Women

O'Brien, W.J.; Shultz, S.P.; Breier, B.H.; Stonehouse, W.; Kruger, R.

Background/Aims: Physical activity (PA) influences predictors of long-term health, particularly cardiorespiratory and metabolic disease risk. This report investigates objectively measured PA levels in healthy, pre-menopausal women participating in the women’s EXPLORE (Examining the Predictors Linking Obesity Related Elements) study.

Methods: In this cross-sectional study, 355 healthy New Zealand (Maori, Pacific, European) women aged 16–45 years, were classified as non-obese or obese (body fat <35% and ≥35%, respectively). Triaxial accelerometers were worn for 7 days to assess levels of sedentary, light, moderate and vigorous PA (0–99, 100–2019, 2020–5998, ≥5999 counts·min^−1^, respectively).

Results: Obese women performed less moderate and vigorous PA than non-obese women. More obese women (71%) performed ≤10 min·week^−1^ vigorous PA than non-obese women (47%), whilst 44% of obese and 16% of non-obese women engaged in no vigorous PA. PA guidelines for moderate PA (150 min·week^−1^) were not met by 43% of obese and 27% of non-obese women. Overall, sedentary behaviour and light PA accounted for 97% of waking time, almost two-thirds of which was sedentary time. Furthermore, more than half of the women were sedentary for >10 h·day^−1^.

Conclusion: Alarmingly, 43% of obese, pre-menopausal New Zealand women failed to meet PA guidelines recommended as beneficial for cardiorespiratory and metabolic health. Furthermore, sedentary behaviour accounted for substantially more time than light, moderate and vigorous PA combined. Our data suggest that there is significant potential to improve long-term health outcomes by spending more time performing PA, especially increasing moderate and vigorous activity levels.

### 2.51. Health Star Rating: Development of a Trans-Tasman Front of Pack Labelling Initiative

Parnell, W.; Hawthorne, P.; Gibbs, M.

Background/Aims: The Health Star Rating (HSR) system was developed by the Australian, state and territory governments in collaboration with industry, public health and consumer groups. The purpose of the system is two-fold: to provide a simple and easy method for consumers to compare the nutritional profile of packaged foods; and provide incentive for reformulation of packaged foods.

Methods: The HSR system uses a star rating scale of 1/2 to 5 stars. The number of stars is determined by an algorithm that considers both the positive and negative aspects of the food product (energy, saturated fat, sugar, sodium, protein, dietary fibre, and the proportion of fruit, vegetable, nut and legume content). The algorithm is based on the nutrient profiling scoring criteria developed by Food Standards Australia New Zealand (FSANZ). The algorithm was tested on approximately 3500 packaged foods across 39 sub-categories. It was modified to enable meaningful ‘within food’ category comparisons and to ensure alignment with the Australian Dietary Guidelines.

Results: Consumer testing was conducted in Australia and New Zealand. HSR positively affected consumers' ability to correctly identify products. New Zealand joined the HSR system following assessment of the system against a set of principles developed by the New Zealand Front-of-Pack Labelling Advisory Group, namely: the need for an interpretive system, meaningful within food categories, readily understood by consumers, and supporting government nutrition policy.

Conclusion: Formative research indicated that the HSR system is able to assist shopper decision making and provide maximum differentiation between foods within categories.

### 2.52. Effects of Legume Addition and Extrusion on the Protein Content and Digestibility of a Wheat-Based Snack

Patil, S.S.; Brennan, C.S.; Mason, S.L.; Brennan, M.A.; Morton, J.D.

Background: Starch, protein and dietary fibres play a vital role in human nutrition. Plant-based foods such as cereals and legumes are rich sources of such nutrients. Many researchers have focused on the importance of legumes (about 20% protein) and cereals (about 50%–60% carbohydrate) in the human diet, as they have potential to meet the nutritional requirement of the increasing global population. This study was undertaken to evaluate the effects of the addition of legume flours into wheat-based extruded snack foods on the protein content and in vitro digestibility of protein of such blends.

Methods: Different proportions (5%, 10%, and 15%) of green pea, yellow pea, chickpea and lentil flour were included in wheat flour based extruded snacks. Protein content was measured by the Dumas method, while in vitro digestibility of protein was determined by a two-step, two enzyme method that simulates digestion. The digested protein was calculated by measuring the difference in the protein content at the beginning and at the end of digestion.

Results: The addition of legumes to wheat-based extruded snacks increased the protein content by 1%–2%. Digestibility of extruded products was significantly higher than raw mixes prior to extrusion. For instance, the protein digestibility of extrudates was 60%–65%, whereas the protein digestibility of raw flour mixes was 28%–35%.

Conclusion: The hot extrusion technique substantially increases protein digestibility of extruded snacks.

### 2.53. The Development and Validation of a Tool to Assess Snacking Habits in Young New Zealand Women

Philipsen, S.; Beck, K.L.; Kruger, R.

Background/Aims: Snacking is common in New Zealand (NZ) women. However, there are few validated tools available to quickly assess foods and food combinations consumed as a snack. This study aimed to develop and validate a Snack Eating Assessment Tool (S-EAT) that assesses the usual snacking habits of NZ women, including the types, combinations and timing of snacks.

Methods: An online self-administered eating habits questionnaire (EHQ) was developed and validated against a 4-day weighed food record (4dwFR) in 108 women, 16–45 years, living in Auckland. The S-EAT is a focused tool within the EHQ used to assess usual snacking habits. Validity was assessed using cross-classification analysis.

Results: Agreement between the S-EAT and 4dwFR for typical snack foods on weekdays and weekends ranged from 70.4% to 92.6% in the morning, although for tea and coffee on weekdays agreement was only 52.8%. In the afternoon agreement on both weekdays and weekends ranged from 61.1% to 86.1%, and in the evening from 70.3% to 87.0%. Typical foods consumed in the morning included fresh fruit, tea and coffee, muesli bars and café drinks; in the afternoon fresh fruit, cheese, yoghurt and crackers; and after dinner tea, coffee, baking and chocolate. “Dairy + grain” was the only commonly consumed food combination (mainly cheese and crackers), with agreement between the EHQ and 4dwFR ranging from 83.3% to 99.0%.

Conclusion: The focused S-EAT is a simple tool that can be used to successfully describe and evaluate the snacking habits of New Zealand women.

### 2.54. Associations between Diet and Diseases and Conditions of the Gastrointestinal Tract of Monogastric and Companion Production Animals

Pluske, J.; Hampson, D.J.; Pethick, D.W.; Kim, J.C.

A number of diseases and conditions of the gastrointestinal tract (GIT) of monogastric animals are associated with the diet that is fed. Increasing evidence suggests that dietary nutrients/components can ameliorate and in some cases, prevent, some diseases and conditions of the GIT. Such a concept has become increasingly important in the intensive livestock industries, e.g., pigs and chickens, where there is increased legislative and consumer scrutiny and in some cases, bans and restrictions, on the use of some antimicrobial compounds used in these production systems. The European Union introduced a ban on the use of sub-therapeutic antibiotics in 2006, which created numerous challenges for these industries in terms of production, health and animal welfare. The predominant reason for this ban, and indeed the pervading reason for legislation and changes in other parts of the world subsequently, is increased resistance by some bacterial pathogens to antimicrobial compounds such as antibiotics and heavy metals (Zn, Cu), which have traditionally been included in feed and (or) water to reduce the prevalence of GIT pathogens.

It is beyond the scope of this paper to discuss the impacts of diet/dietary components on every disease and condition recognised in the GIT of these animals. Nevertheless, there are a number of economically significant diseases and conditions of pigs and chickens whose aetiology is embedded with a strong dietary influence. In pigs, diseases and conditions such as swine dysentery (SD), post-weaning diarrhoea (PWD), gastric ulcers and Salmonellosis are known to be affected by specific dietary nutrients and (or) components, or by the method of feed processing before being offered to the animals. Similarly and in chickens (both meat and egg birds), diseases and conditions such as necrotic enteritis (NE), wet litter, Salmonellosis, avian intestinal spirochaetosis, and Campylobacteriosis have a strong dietary influence. In companion animals, the nature of the diet(s) fed can have profound influences on laminitis in horses and small intestinal bacterial overgrowth in dogs, for example.

Research conducted by our group, amongst others, has shown that some of these diseases and conditions can be controlled and (or) prevented using specific nutritional manipulation, sometimes in the absence of antimicrobial compounds. For example, the use of lower protein diets and manipulation of type and content of the dietary fibre can impact upon the incidence of PWD and SD, whilst in meat chickens, the use of certain grains and carbohydrate fractions is known to influence NE. The influence of the diet on fermentation characteristics in the large intestine can also have associations with stool form and osmotic diarrhea. In this regard, positive aspects of some fermentative substrates (e.g., resistant starch, oligosaccharides, soluble fibre) for human nutrition often have negative consequences for monogastric animals from a production, faecal form, and (or) disease perspective.

Interactions between nutrients, components and (or) ingredients and the GIT to affect a range of diseases and conditions are complex. In a rapidly changing regulatory and consumer landscape, the monogastric production and companion animal industries face new challenges related to production, animal health and welfare.

### 2.55. Impact of Lactation Patterns on the Hormonal Composition of Human Breast Milk

Pundir, S.; Thorstensen, E.B.; Linderborg, K.M.; Lagström, H.; Fraser, K.; Roy, N.C.; Cameron-Smith, D.

Background/Aims: In addition to providing nutrients, human breast milk (HBM) delivers non-nutritive bioactive elements, including glucocorticoids (GCs; cortisol and cortisone) to breastfeeding (BF) infants. Milk-borne GCs are important regulators of stress-mediated responses and may influence infant physiological and psychological development. Many factors are known to influences maternal stress, and hence the GC composition of breast milk. However, little is known about how partial BF (which affects milk volume, and frequency and regularity of feeding) may influence GC hormone levels in HBM. We hypothesised that different BF patterns have a major impact on the composition and volume of milk-borne GCs. We aimed to determine the variation of GC steroid hormone concentrations in HBM related to different breastfeeding patterns (exclusive vs. partial BF).

Methods: Samples were obtained from lactating mothers participating in the Finnish STEP study (Steps to the Healthy Development and Well-being of Children). GC concentrations were measured using liquid chromatography-tandem mass spectrometry (LC-MS/MS) in 652 samples adjusting for infant sex. Data on BF patterns and infant gender were obtained using self-reported diaries and hospital records.

Results: Cortisone (mean 9.5 ng/mL) and cortisol (mean 7.5 ng/mL) were detected in all samples. Infant gender had no impact on GC concentrations. Furthermore, no difference in GC levels was observed between the exclusive and partial BF groups.

Conclusion: GC levels in HBM are not associated with variation of BF pattern or with infant gender. Further analysis is ongoing to determine the impact of maternal health and nutritional intake on GC levels in HBM.

### 2.56. Glycated Albumin Does Not Correlate with Postprandial Markers of Glycaemia in Normoglycaemic Young Adults

Reynolds, A.N.; Venn, B.; Duncan, A.; Kruimer, D.; Mann, J.

Background/Aims: There is clinical and research value in a marker reflective of glycaemic control over the previous 2–3 weeks. Glycated albumin is one such potential marker. To determine the ability of glycated albumin in the initial identification of impaired glucose tolerance we assessed glycated albumin against other glycaemic values and markers of health status in a population of normoglycaemic young adults.

Methods: Eighty four young adults of normal glucose tolerance underwent a 2-h 50 g oral glucose tolerance test. Glycated albumin values were correlated against fasted (FPG) and 2 h post load plasma glucose measures (2hPG), incremental area under the curve (iAUC), glycaemic range, body mass index (BMI) and C-reactive protein (CRP).

Results: When adjusted for age and sex, glycated albumin was inversely correlated with BMI (*r* = −0.25, *p* = 0.03). No significant correlations existed for glycated albumin and postprandial measures of glycaemia. BMI and CRP values correlated in this population (*r* = 0.30, *p* < 0.01) as has been reported previously.

Conclusion: Glycated albumin was not associated with postprandial markers of glycaemia in a population of normal glucose tolerant young adults. Further research is required to determine its potential in identifying impaired glucose tolerance.

### 2.57. Dietary Fatty Acids, Plasma Phospholipid Fatty Acids and Associations with Blood Lipids in Healthy South Africans from the PURE Study

Richter, M.; Baumgartner, J.; Wentzel-Viljoen, E.; Smuts, C.M.

Background: Plasma phospholipid EPA and DHA have been used as biomarkers of intake. Previously, we found dietary alpha-linolenic acid (ALA) intake was positively associated with triglycerides in men. Therefore, we investigated plasma phospholipid fatty acids (FA) composition in healthy South Africans and explored associations between dietary FA intake and plasma FA composition, as well as associations between plasma FAs and blood lipids.

Methods: A cross-sectional analysis within the Prospective Urban Rural Epidemiology (PURE) baseline study of healthy subjects (35–70 years) in South Africa. Dietary data were collected. Blood lipid and plasma total phospholipid FA analyses were performed on a random subsample (*n* = 716).

Results: Mean plasma DHA ranged between 3.45%–5.43%. We found a positive correlation between dietary ALA and plasma DHA (men *r* = 0.33, *p* < 0.001; women *r* = 0.30, *p* < 0.001), which was stronger than the association between dietary and plasma DHA (men *r* = 0.21, *p* < 0.001; non-significant in women). Plasma phospholipid DHA was positively associated with triglycerides in men (β = 0.410, *p* < 0.001) and women (β = 0.379, *p* < 0.001).

Conclusion: Even though previously reported median dietary EPA + DHA intake in this population was below recommendations, mean plasma DHA was relatively high compared to healthy individuals in other studies. The positive correlation between dietary ALA and plasma DHA, together with the association between plasma DHA and triglycerides, is in line with the finding from our previous study, indicating towards an efficient conversion of ALA to DHA. These results suggest that plasma phospholipid FAs shouldn’t be used in isolation as biomarkers for intake in epidemiology without assessing dietary intake.

### 2.58. Habitual Sweet Food and Beverage Intake Is Influenced by Perception of Sweet Taste Intensity

Rivers, S.; Jayasinghe, S.N.; Kruger, R.; Breier, B.H.

Background/Aims: Sugar consumption creates pleasure, but excessive sugar consumption leads to weight gain and is a key driver of obesity. This study aims to assess sweet food and beverage intake and behaviours and how they may be explained by perceived sweet taste intensity.

Methods: Women (*n* = 45), aged 20–40 years, were recruited for this cross-sectional study. A non-quantitative sweet food-food frequency questionnaire (SF-FFQ) was developed to assess intakes. The three-factor eating questionnaire (TFEQ) was used to assess eating behaviour. Perception of the sweet taste intensity of glucose concentrations (125 mM, 250 mM, 500 mM, 1000 mM) was rated (0–100) on a modified general Labelled Magnitude Scale.

Results: Frequency of daily intake was reported as daily frequency equivalents (DFE). The mean DFE of occasional sweet food was high (4.23 ± 2.29), with baking and sweets intake especially high (1.20 ± 0.83). Women with a self-reported “sweet tooth” consumed significantly higher DFE of baking (*p* = 0.04), chocolate (*p* = 0.03) and soft lollies (*p* = 0.04) than women with no “sweet tooth”. Chocolate DFE was significantly higher in women who experienced regular food cravings compared to women who did not (*p* = 0.00). Higher sweet food consumption was correlated with less sensitivity to 1000 mM glucose (*r* = −0.35, *p* = 0.02). Women who preferred sweet snacks were less sensitive to 1000 mM glucose than those who preferred savoury snacks (*p* = 0.04).

Conclusion: Women with a lower sensitivity to sweet taste were more likely to consume more sweet food. Our data suggest that sweet taste intensity perception plays an important role in habitual sweet food and beverage intake.

### 2.59. Bioactive Fractioning of Ganoderma Lucidum Triterpenoids Show Cell Specific Effects on Cancer Cell Growth

Ruan, W.; Popovich, D.

Background/Aims: *Ganoderma lucidum* is an edible mushroom with a long history of use in traditional Chinese medicine. The mushroom has been associated with a variety of pharmaceutical activities and it is thought that the triterpenoids are the main active group with chemo preventative properties. The aim was to assess the effect of purified triterpenoids on cultured cancer cell growth

Methods: We used a bioactive fraction protocol to identify six triterpenoids by ESI-MS and NMR which were tested in three distinct cultured human cancer cell lines.

Results: Six compounds (ganodermanontriol, ganolucidic acid E, lucidumolA, 7-oxo-ganoderic acid Z, 15-hydroxy-ganoderic acid S, ganoderic acid DM) reduced cultured cancer cell growth in three cancer cell lines colon (Caco-2), the liver (Hep-G2) and the cervix (HeLa) to varying degrees. LC50 ranged from 20.87–84.36 µM. Ganodermanontriol had the greatest inhibitory effect in HepG2 cells with an LC50 of 20.87 ± 1.48 µM, while 15-hydroxyl-ganoderic acid S exhibits the most cytotoxicity in HeLa and Caco-2 cells with LC50s of 21.17 ± 1.81 µM and 30.38 ± 1.44 µM respectively. Apoptotic cell death was observed for all compounds tested in Hela cells with 15-hydroxy-ganoderic acid S showing the greatest sub-G1 cells (22%) in cell cycle analysis and apoptotic positive cells in TUNEL assay (43.03%). These results were confirmed by annexin-V staining. None of the compounds induced apoptosis in Hep-G2 cells.

Conclusion: Bioactive triterpenoids from *Ganoderma lucidum* induce apoptosis and likely trigger different cell death pathways which are dependent on tissue type and function.

### 2.60. A Short Dietary Assessment Tool in Children

Saeedi, P.; Skeaff, S.A.; Wong, J.E.; Skidmore, P.M.L.

Background/Aims: Dietary habits formed in childhood can have a significant impact on their health later in life. It is therefore essential to measure children’s food intake accurately. The aim of this study was to assess the reproducibility and relative validity of a qualitative 28-item food frequency questionnaire (FFQ), namely the Physical activity, Exercise, Diet And Lifestyle Study (PEDALS FFQ) in 9–10 year-old children.

Methods: Fifty children from three schools in Dunedin, New Zealand, completed the PEDALS FFQ twice and also a four day estimated food diary (4DEFD) within a two-week interval. Intraclass correlation coefficients (ICC) and Spearman’s correlation coefficients (SCC) were used to determine reproducibility and relative validity of the FFQ, respectively. Weekly intakes were estimated for each food item and aggregated into 23 food items/groups.

Results: More than half of the food items/groups (52.2%) had an ICC higher than 0.5. The median SCC between FFQ administrations was 0.66 (ranging from 0.40 for processed meat to 0.82 for sweets and non-dairy drinks). For validity analyses, 70% of food items/groups had a SCC ≥0.3. Cross-classification analysis between the first FFQ and 4DEFD for ranking participants into thirds showed breakfast cereals (54.0%) had the highest agreement and pasta (34.0%) the lowest.

Conclusion: The results indicate that the PEDALS FFQ is useful for ranking subjects according to food group intake. The low respondent burden and relative simplicity of this FFQ means it is suitable for use in large cohort studies of 9–10 year-old children in New Zealand.

### 2.61. The Use of RSM to Produce a Nutritious Fruit Leather Product

Savage, G.; Feng, P.

Background/Aims: Manufacture of a new nutritious product from locally produced green kiwifruit and blackcurrant purée needs careful consideration of the optimum mix of ingredients and optimum processing conditions to produce a nutritious and stable fruit leather.

Methods: Response surface methodology (RSM) efficiently identifies, from a small number of experiments, a mix of purées with added sugar and pectin. RSM methods were then used to optimise the combination of drying time and temperature to give maximum responses for colour retention, ascorbic acid content and suitable texture.

Results: The optimum combination of the ingredients was 80.98% green kiwifruit, 9% blackcurrant purée with 10% sucrose and 0.02% added pectin. 16 h drying at 65.4 °C produced a tasty product.

Conclusion: The final product contained 79% soluble carbohydrates, 4% protein, 7.5% fibre and excellent colour. Moisture and pH content suggested that the product would be stable in storage.

### 2.62. Oxalate Content of Green Juices Produced by Two Different Methods

Savage, G.; Vanhanen, L.

Background/Aims: Green juices are promoted as a rich source of nutrients and vitamins. Unfortunately green juices are commonly made from green leafy vegetables that can contain high levels of oxalates. Several cases of acute oxalate nephropathy have been attributed to the consumption of green juices.

Methods: Two green juices were prepared, one using 20% spinach and another otherwise identically proportioned juice contained 40% spinach. Both the low and high spinach containing green juices were prepared using a masticating juicer (MJ) which produced a clearer thinner juice and a high speed blender (HSB), produced a thicker juice. Total, soluble and insoluble oxalate contents of the four juices were determined by HPLC.

Results: Overall there was no significant difference between the soluble oxalate content in the low spinach recipe (77.7 and 97.7 mg/100 g FW, HSB and MJ respectively). However, the high spinach juice produced by the MJ had a significantly higher amount of soluble oxalate (364.1 mg/100 FW) compared to the HSB blender (275.3 mg/100 g FW).The ratio of soluble to total oxalate in the different types of juices was similar, 45% for the low spinach juice and 46% and 66% and 74% for the high, HSB and MJ, respectively. The consumption of 200 mL of any of the juices would lead to the consumption of between 155–728 mg of soluble oxalate.

Conclusion: Different recipes and different types of juicers give very different oxalate contents in the resulting juices.

### 2.63. Exploring the Dietary Intake and Patterns of Young New Zealand European Women with Different Body Composition Profiles

Schrijvers, J.; McNaughton, S.; Beck, K.L.; Stonehouse, W.; Kruger, R.

Background/Aim: Analysing dietary patterns provides an alternative measure to investigate dietary habits related to excess adiposity. The aim of this study is to investigate dietary intakes and patterns of New Zealand European (NZE) women with different body composition profiles, participating in the women’s EXPLORE (Examining the Predictors Linking Obesity Related Elements) study.

Methods: Post-menarche, pre-menopausal NZE women (16–45 years) (*n* = 231) completed a validated 220-item, self-administrated, semi-quantitative food frequency questionnaire (FFQ) assessing dietary intake over the previous month. Quetelet’s BMI was calculated (kg/m^2^) from height and weight measurements; body fat percentage (BF%) was measured using air displacement plethysmography (BodPod). Dietary patterns were identified using principal component factor analysis. Associations between dietary patterns, age, BMI and BF% were investigated.

Results: Four dietary patterns were identified: P1: high fat and sugar; P2: meat and energy-dense; P3: fruit and vegetable; P4: meat alternatives, which explained 6.9%, 6.8%, 5.6% and 4.8% of variation in food intake, respectively. Mean ± SD age (30.0 ± 8.3 years) (*p* = 0.048) and BMI (26.4 ± 26.7) (*p* = 0.036) were significantly associated with women loading highest (tertile 3) in P2. No significant associations were found with BF%. All patterns met the estimated average requirements for all nutrients. Mean ± SD percentage of energy intake for carbohydrate (41.9% ± 7%) was below and for saturated fat (13.9% ± 3.5%) above the acceptable macronutrient distribution range.

Conclusion: Dietary patterns in NZE women may not explain all the differences seen between individual body compositions. Other factors such as macronutrient distribution and portion size may play a role.

### 2.64. The Effect of Different Fatty Acids on Markers of Inflammation in Hypothalamic Neurons

Sergi, D.; Williams, L.; Kahn, D.E.; Drew, J.E.

Background/Aims: A high-fat diet (HFD) is known to cause inflammation in the hypothalamus of rodents. Microglia, the resident immune-reactive cells and astrocytes are involved in this process. However, it is unclear whether these cells initiate the response, or are activated as a result of inflammation in neurons. Our aim was to investigate the effects of different fatty acids on a marker of inflammation (IL-6) in hypothalamic neurons.

Methods: We treated hypothalamic neurons mHypoE-N42 (N42) with different fatty acids and mixtures of fatty acids at physiological concentrations (200 µM) and measured *IL-6* gene expression using semi-quantitative Real-Time PCR. Differences between groups were tested using Student’s *t*-tests.

Results: Palmitic acid (16:0) and decanoic acid (10:0) upregulated *IL-6* (*p* < 0.001) whiledocosahexaenoic acid (DHA) (22:6 *n*-3) and oleic acid (18:1 *n*-9) downregulated *IL-6* (*p* < 0.001)*.* Eicosapentaenoic acid (EPA) (20:5 *n*-3), lauric acid (12:0) and octanoic acid (8:0) had no effect on *IL-6* expression. When administered in combination with palmitic acid, both EPA and lauric acid were able to reduce PA induced inflammation (*p* < 0.01 and *p* < 0.001 respectively).

Conclusion: This study shows that fatty acids varying in chain length and degree of saturation elicit different outcomes in expression of the inflammatory marker *IL-6* in neurons and confirm the well-documented anti-inflammatory effect of oleic acid and the polyunsaturated fatty acids, DHA and EPA, revealing differential effects, with DHA inhibiting inflammation and EPA reverting pre-existing inflammation.

### 2.65. Breastfeeding Practices of Preterm Babies Admitted to the Special Care Baby Unit at Whangarei Hospital, New Zealand

Share, A.N.; McNab, M.; Gammon, C.S.; Conlon, C.A.

Background/Aim: Maternity units within Northland DHB are Baby Friendly Hospital Initiative accredited and report high exclusive breastfeeding rates (>90%). However, delayed physiological development of preterm babies, which can impact gastrointestinal maturity and feeding skills, can make breastfeeding problematic. This study aimed to investigate breastfeeding practices of preterm babies admitted to the Special Care Baby Unit (SCBU) at Whangarei Hospital.

Methods: Retrospective data on breastfeeding practices was collected from medical notes of preterm babies (*n* = 100) admitted to SCBU for a minimum of 3 days between January 2013 and March 2014.

Results: The median age of mothers was 27 years (range 17–43) and 56% identified as NZ Māori. The median (25th, 75th percentile) age of the babies was 35 weeks gestation (33, 36) and 57 were male. Fourteen babies were born extremely premature (≤32 weeks’ gestation) and 86 were of moderate to late prematurity (>32 to <37 weeks’ gestation). Median length of SCBU admission was 14 days. Breastfeeding was initiated by 83% of the mothers. Seventy-six babies received enteral feeding, with 45 commencing on expressed breastmilk. On discharge, 73% were receiving some breastmilk with 67% exclusively breastfeeding.

Conclusion: Preterm babies admitted to SCBU had high rates of breastfeeding initiation and the majority were receiving breastmilk at discharge. Nevertheless, there is still a gap between these rates and overall exclusive breastfeeding rate reported by Northland DHB. Future studies need to determine how mothers of preterm babies can be further supported to establish exclusive breastfeeding.

### 2.66. Development of a Salt Reduction Model for New Zealand

Shields, E.; Eyles, H.; Webster, J.; Ni Mhurchu, C.

Background/Aims: New Zealand has committed to the United Nations target of a 30% relative reduction in population dietary sodium intake by 2025. We developed a salt reduction model for New Zealand to determine the decreases required in sodium content of packaged foods to reduce population salt intake from the current 8.4 g/day to the optimal World Health Organization target of 5 g/day.

Methods: We adapted the methods of the successful UK strategy. Nationally-representative food sales data were linked with branded food composition information to determine the average contribution of major food categories to total salt consumption. Salt consumed at the table and in foods away from the home was estimated from National Nutrition Survey data. Target values resulted in the mean sodium content of each food category meeting an overall population salt intake of 5 g/day.

Results: Assuming a 40% reduction in added salt and the sodium content of foods consumed away from the home, we calculated that a 36% reduction (1.6 g salt or 628 mg sodium) from packaged foods would reduce the population’s salt intake to 5 g/day. Percentage reductions included 18% for white bread, 27% for hard cheese, 42% for sausages, and 47% for ready to eat cereals.

Conclusion: The estimated reductions required in the sodium content of New Zealand foods should be used to inform formal targets for food manufacturers to drive reformulation across the national food supply. The targets should be implemented as part of a government-led strategy to reduce population salt intake.

### 2.67. Through the Eyes of a Child: Environmental Interventions to End Childhood Obesity

Signal, L.

Ending childhood obesity is the ambitious challenge of the recent World Health Organization Commission. In this presentation we explore, through the eyes of a child, a range of environmental interventions to end childhood obesity. We present initial findings from the innovative *Kids’Cam* project where 169 Year 8 children from the Wellington region wore an automatic camera for four days and filmed their world. This, and other research presented, was undertaken by staff of the Health Promotion & Policy Research Unit at the University of Otago and our collaborators, including colleagues at the National Institute for Health Innovation at the University of Auckland. The presentation will focus on the school food environment, the junk food marketing environment and the value of food taxes and food security.

### 2.68. Fostering Environmental Education within a University Campus Food Outlet Environment

Slobbe, C.; Mirosa, M.; Thomson, C.

Background: University foodservices have the potential to foster environmental education. Understanding the perspectives of foodservice staff is critical for engaging staff to implement environmental education in universities.

Methods: Q methodology, a mixed methods approach to understanding dominant shared sets of attitudes, was used to determine a range of perspectives on environmental education. The study design integrates two phases. The first phase involves preliminary interviews with foodservice stakeholders and a card sorting activity. The second phase will convert the results of the sorting activity into a survey to determine the prevalence of perspectives in a wider national population of university food service staff.

Results: Phase one interviews with two university academics and three university foodservice stakeholders were conducted to develop an assortment of statements pivoting around the issue of environmental education. The interviews as well as perspectives from secondary data, grey and academic literature were used to create a table of 92 statements. Thematic analysis of preliminary results showed saturation of positive views, where similar statements occur in interviews. Further phase one interviews (*n* = 40) with foodservice staff will identify groups of viewpoints called factors.

Conclusion: The results provide perspectives of foodservice staff about environmental education in their workplace. Future research will determine the prevalence of viewpoint groups across university foodservice staff nationwide. The study will help provide a model to guide universities in making preliminary steps to integrate environmental education into their foodservice operations.

### 2.69. Types of Sweetened Beverages Consumed and Contribution to Energy Intake in New Zealand Adults

Smith C.; Parnell, W.R.

Background/Aims: Reducing the consumption of sweetened beverages is a popular health promotion focus. The aim of this research was to examine the prevalence of consumption, total sugar intake and percent of daily energy from sweetened beverages by sex and age group in New Zealand (NZ).

Methods: The 2008/09 NZ Adult Nutrition Survey was a cross-sectional survey of a national sample (15+ years, *n* = 4721). A computer based multiple-pass 24-h diet recall was used. All foods consumed were coded into main food groups and sub-food groups including beverages.

Results: In total 17.5% reported a sweetened beverage. The most frequently reported (excluding tea or coffee) were soft drinks (7.5%) and fruit juice (3.1%). Males were more likely to report soft drinks compared to females (*p* < 0.001). Daily energy from soft drinks was highest among those 15 to 18 years (3.6%) and lowest among adults 71 years and over (0.4%). Among consumers the grams of sugar per day from sweetened beverages for young adults (<30 years) was nearly double that of other age groups.

Conclusion: Sweetened beverage consumption was more prevalent among younger age groups. Reducing sweetened beverage health promotion activities should focus on young adults, particularly males.

### 2.70. Okara as Flour Substitute for the Development of Nutritious Functional Snack Food

Tan R.C.H.; Prabha, S.

Background: Okara or soybean pulp is a by-product from soymilk production and is high in fibre and protein. Currently okara is not utilized and is classified as agro-waste. This study was conducted to develop nutritious baked doughnuts using okara. Okara was partly used as a substitute ingredient for flour in the manufacture of the doughnuts.

Methods: A market survey was conducted to determine consumer attitude and awareness of okara products. For the product development, four formulations were developed; S1, S2, S3 and S4 with 0%, 15%, 30% and 45% okara flour, respectively. Sensory evaluation (nine-point hedonic test) and proximate analysis of the doughnuts were conducted.

Results: In the market survey, it was determined that health and nutrition (*r* = 0.50; *p* < 0.01) played the biggest role in determining attitude and awareness towards okara food products, followed by price and convenience (*r* = 0.40; *p* < 0.01), weight control (*r* = 0.40; *p* < 0.01), and sensorial appeal (*r* = 0.26; *p* < 0.01). From the hedonic test, five attributes were taken into account: sweetness, texture, flavor, aroma and overall likeability. Sample S3 with 30% okara flour scored the highest overall likeability (6.36 ± 1.9). For the proximate analysis of the four formulations, the addition of okara flour in the doughnuts significantly (*p* < 0.05) increased the protein, fibre, moisture, lipid and ash contents.

Conclusion: Taking into account both sensory evaluation and proximate analysis, sample S3 with 30% okara flour has the potential to be successfully commercialized as a nutritious product in the functional food market.

### 2.71. Understanding the Bowel Microbiota

Tannock, G.

The human colon is home to trillions of bacterial cells that form a community known as the microbiota. Bacterial communities form in the bowel early in life and undergo compositional developments that are driven by the nutrition of the child. First studied more than 100 years ago, the composition of the microbiota of the child during the exclusively milk-fed period is relatively simple, differing according to whether human milk or cow’s milk formula is fed to the baby. Once solid foods are introduced, the composition of the microbiota becomes more complex due to the introduction of food components, mostly plant polymers, that are undigestible to humans, but which provide substrates for bacterial growth in the colon. Thus, some dietary components resemble garden fertilisers; they promote the growth of particular bacterial groups that are able to hydrolyse the polymeric substances, as well as those that can ferment the hydrolytic products. Interactions between different kinds of bacteria result in consortia of species which process plant carbohydrates, producing short chain fatty acids. The influence of dietary components on microbiota composition has, in recent decades, been inferred from the analysis of DNA sequences that are informative of bacterial taxonomy (16S rRNA gene), as well as that of the biochemical capacity of the community as a whole (the metagenome). The types of bowel bacteria that occur commonly in human faeces, representing the main metabolic groups in the colon, have been cultivated. High throughput DNA sequencing studies that reveal the complexity and individuality of microbiotas provide platforms to study interactions between bowel bacteria under laboratory conditions. By understanding the bowel ecosystem and its food-bacteria matrices, we may be able to enhance, through nutritional interventions, the establishment of specific consortia in the bowel, and alleviate the symptoms associated with some human conditions and diseases.

### 2.72. A Systematic Review of Studies Investigating the Anthelmintic Effect of Datepalm Fruit

Wagner, K.

Aging is the inevitable accumulation of physical changes in organisms over time, which progressively increases the risk of disease and mortality.

Advancing adult age is associated with profound changes in body composition. One of the most prominent of these changes is sarcopenia, defined as the age-related loss in skeletal muscle mass, which results in decreased strength and aerobic capacity and thus functional capacity. Sarcopenia is also closely linked to age-related losses in bone mineral content, basal metabolic rate and increased body fat content.

Skeletal muscle is a highly malleable tissue, whereby muscle mass is determined by a fine-tuned network of muscle growth and degradation pathways. While the activation of the phosphoinositide 3-kinase (PI3K)/Akt pathway by insulin-like growth factor-1 (IGF-1) leads to muscle hypertrophy, its inhibition by myostatin, a member of the transforming growth factor-β (TGF-β) family, generally lead to muscle atrophy and inhibits muscle differentiation. Additonally other TGF-β family members such as activin A and growth differentiation factor-15 (GDF-15) seem to have a negative impact on skeletal muscle growth.

With these aspects in mind it is not surprising that many of these molecules are suggested as blood-based biomarkers of aging. In older women, IGF-1 correlates negatively with age and positively with muscle mass while GDF-15 is positively associated with age and negatively with muscle mass. Contrasting results have been detected for serum myostatin levels which are negatively correlated to muscle mass in male patients with chronic obstructive pulmonary disease.

With regard to nutrition total energy intake decreases with the aging process emphasising the importance of a nutrient-dense diet. Furthermore as the organs lose function, impairments in bioavailability and activation of various nutrients such as vitamin B12 or D begin to occur. In this case it is important to assess nutrient status using biomarkers in urine, plasma or sometimes very specifically at cellular level such as for folic acid.

Further mechanisms that contribute to the aging process and the development of chronic, age-associated diseases include increased levels of DNA damage, genotoxicity, oxidative stress, and shorter telomeres. These latter parameters also have a close link to nutrient status and are altered when storage levels are low.

This talk summarizes nutritional and muscular biomarkers for aging, linking them to changes based on lifestyle intervention programs in elderly and specifically focusing on the cellular and molecular level changes. Finally this talk will address longevity and changing biomarkers of elderly living beyond average life expectancy compared to subjects below life expectancy.

### 2.73. Exploring Dietary Patterns during Pregnancy in a Multi-Ethnic Society Context

Wall, C.R.; Grant, C.C.; Bandara, D.K.; Atoea-Carr, P.E.; Morton, S.M.B.; Gammon, C.S.

Background/Aims: Exploration of maternal dietary pattern associations within a multi-ethnic society context has been limited. The aim of this study was to describe the dietary patterns of pregnant women from ‘Growing Up in New Zealand’, a large ethnically-diverse cohort, and to investigate associations between these patterns, ethnicity and birthplace.

Methods: During 2009 and 2010 5664 women completed a semi-quantitative food frequency questionnaire prior to childbirth. Principal component analysis was used to describe dietary patterns, and multivariable regression analyses to determine associations.

Results: Self-prioritised maternal ethnicity was 56% European, 13% Māori, 13% Pacific, 14% Asian and 4% other ethnicities. 35% were born outside New Zealand (NZ). Four distinct dietary components were extracted: ‘Junk’ and ‘Health conscious’, both being associated with being born in NZ and not being of Asian ethnicity; ‘Traditional/White bread’ which showed no association with place of birth; and ‘Fusion/Protein’ associated with being born outside NZ and most strongly with Asian ethnicity. ‘Junk’ and the ‘Traditional/White bread’, were associated with being of Pacific or Māori ethnicity. The two healthier patterns were associated with increased maternal age, better self-rated health, lower pre-pregnancy BMI and not smoking; the two unhealthier patterns were associated with decreased age, lower education levels, and smoking.

Conclusion: A greater understanding of the influence of migration and ethnicity on dietary patterns in association with other socio-demographic factors could allow for more targeted strategies to support good nutrition during pregnancy.

### 2.74. The Effect of Whey Protein on GLP-1 Secretion from Human Gut Explants Compared with STC-1 Cells

Williams, L.; Song, H; Crosbie, L.; Morris, A.C.; Hempseed, G.; Drew, J.E.; Bunting D.; Macluskie G.

Background/Aims: Whey protein increases the secretion of GLP-1 from the gut. We tested whey protein and digests on GLP-1 secretion from human gut explants, known to contain GLP-1 secreting cells and on mouse derived STC-1 cells, frequently used to study GLP-1 secretion.

Methods: Human colon tissue wasfrom Biopta (Glasgow, UK). Samples were cultured with a negative control: Hank’s buffer, a positive control: meat peptone, undigested whey protein, partially digested whey protein (total protein, <1 KDa and >1 KDa fractions) fully digested whey protein (total protein, <1 KDa and >1 KDa fractions) and amino acids matched to whey protein, all at 25 mg/mL. Samples were incubated for 2 h and total GLP-1 measured using a total GLP-1 ELISA kit. Differences were tested using Student’s *t*-tests.

Results: Meat peptone stimulated the greatest secretion of GLP-1 from the explants compared to negative control (*p* < 0.001), as expected, while undigested whey and the amino acid mixture resulted in no significant increase in GLP-1 secretion. Partial and full whey protein digests all significantly stimulated GLP-1 secretion to approximately the same extent compared to control (*p* < 0.05). This was in contrast to experiments on STC-1 cells where the undigested whey protein resulted in a significant increase (*p* < 0.05) in GLP-1 secretion compared to digests.

Conclusions: These results indicate that the use of STC-1 cells must be treated with caution when making assumptions regarding the efficacy of nutrients to elicit a GLP-1 response in humans.

### 2.75. Prevalence of Vitamin D Insufficiency in Taiyuan City and Its Relationship with Risk for Metabolic Syndrome

Yan, X.; Thomson, J.; Weber, J.; Coad, J.

Background: Emerging evidence suggests vitamin D deficiency might be associated with incidence of Metabolic Syndrome (MetS). There are concerns about the increasing prevalence of MetS in China, particularly in younger people.

Aims: To investigate the prevalence of vitamin D deficiency and its possible association with MetS in non-manual urban dwellers in a Northern Chinese city known to be affected by industrial pollution.

Methods: 200 apparently healthy participants attending the Health 100 Check-up Center in Taiyuan City, during the winter months, for their annual health appointment were asked questions about lifestyle and had their serum vitamin D levels and markers for MetS measured.

Results: 78% of participants had serum 25-hydroxyvitamin D (25(OH)D) levels <50 nmol/L; below the cut-off for sufficiency. Serum 25(OH)D levels in female participants (median 32.7 nmol/L, interquartile range (IQR): 25.80, 43.80) were lower than in male participants (median 44.0 nmol/L; IQR: 32.30, 55.40). Serum 25(OH)D levels were lower in women under 40 years old (29.25 nmol/L; IQR: 24.05, 40.85) compared to women over 65 years old. The prevalence of MetS was 29.9%. Multiple linear regression analysis identified increased fasting glucose to be significantly associated with vitamin D status (*p* < 0.05) but there was no other association between vitamin D status and other markers of MetS.

Conclusion: Vitamin D insufficiency was highly prevalent, particularly in younger women. Female gender and fasting glucose levels were significantly associated with vitamin D insufficiency.

## 3. Affiliations

- Abdullah, W.A. Universiti Sains Islam Malaysia, Kuala Lumpur, Malaysia
- Addnan, F. Universiti Sains Islam Malaysia, Kuala Lumpur, Malaysia
- Agnew, M., AgResearch Ltd., Palmerston North, New Zealand; Nutrigenomics NZ, Auckland, New Zealand
- Aitchison, C., Department of Human Nutrition, University of Otago, Dunedin, New Zealand
- Alexander, D.L., Ministry for Primary Industries, Wellington, New Zealand
- Allen, J., Waitemata District Health Board, Auckland, New Zealand
- Atoea-Carr, P.E., Centre for Longitudinal Research—He Ara ki Mua, The University of Auckland, Auckland, New Zealand
- Awan, T., School of Food and Nutrition, Massey University, Palmerston North, New Zealand
- Baggett, F., School of Food and Nutrition, Massey University, Auckland, New Zealand
- Bandara, D.K., Centre for Longitudinal Research—He Ara ki Mua, The University of Auckland, Auckland, New Zealand
- Barker, M., Medical Research Council Lifecourse Epidemiology Unit, University of Southampton, Southampton, United Kingdom
- Baumgartner, J., Centre of Excellence for Nutrition, North-West University, Potchefstroom, North West, South Africa
- Beck, K.L., School of Food and Nutrition, Massey University, Auckland, New Zealand
- Black, K., Department of Human Nutrition, University of Otago, Dunedin, New Zealand
- Blatchford, P., Plant and Food Research Limited, Palmerston North, New Zealand
- Borman, B., Centre for Public Health Research, Massey University, Wellington, New Zealand
- Boss, A., Faculty of Medical and Health Sciences, University of Auckland, Auckland, New Zealand
- Bozonet, S., Centre for Free Radical Research, University of Otago, Christchurch, New Zealand
- Bradbury, K.E., Nuffield Department of Population Health, Oxford University, Oxford, United Kingdom
- Breier, B.H., School of Food and Nutrition, Massey University, Auckland, New Zealand
- Brennan, C.S., Faculty of Agriculture and Life Sciences, Lincoln University, Lincoln, New Zealand
- Brennan, M.A., Faculty of Agriculture and Life Sciences, Lincoln University, Lincoln, New Zealand
- Brough, L., School of Food and Nutrition, Massey University, Palmerston North, New Zealand
- Brown, R.C., Department of Human Nutrition, University of Otago, Dunedin, New Zealand
- Bunting, D., Weipers Centre, Glasgow, United Kingdom
- Butts, C., Plant and Food Research, Palmerston North, New Zealand
- Cairncross, C.T., School of Food and Nutrition, Massey University, Auckland, New Zealand
- Camargo Jnr, C., Massachusetts General Hospital, Boston, USA
- Cameron-Smith, D., Liggins Institute, University of Auckland, Auckland, New Zealand
- Carr, A., Centre for Free Radical Research, University of Otago, Christchurch, New Zealand
- Casale, M., School of Food and Nutrition, Massey University, Palmerston North, New Zealand
- Chepulis, L., Waiariki institute of Technology, Rotorua, New Zealand
- Coad, J., School of Food and Nutrition, Massey University, Palmerston North, New Zealand
- Congalton, D., Gravida: National Centre for Growth and Development, University of Auckland, Auckland, New Zealand
- Conlon, C.A., School of Food and Nutrition, Massey University, Auckland, New Zealand
- Crosbie, L., Rowett Institute of Nutrition and Health, Aberdeen, United Kingdom
- Daniels, L., Department of Human Nutrition, University of Otago, Dunedin, New Zealand
- Davidson, R., Department of Human Nutrition, University of Otago, Dunedin, New Zealand
- Davies, B., Department of Human Nutrition, University of Otago, Dunedin, New Zealand
- Davison, B., Department of Human Nutrition, University of Otago, Dunedin, New Zealand
- de Bray, J.G., School of Food and Nutrition, Massey University, Auckland, New Zealand
- Deo, N.N., School of Food and Nutrition, Massey University, Auckland, New Zealand
- Dinnan, H., Plant and Food Research, Palmerston North, New Zealand
- Drew, J.E., Rowett Institute of Nutrition and Health, Aberdeen, United Kingdom
- Duncan, A., Department of Human Nutrition, University of Otago, Dunedin, New Zealand
- Edmonds, J.C., Food Science and Risk Assessment Directorate, Ministry for Primary Industries, Wellington, New Zealand
- Eickstaedt, M., School of Food and Nutrition, Massey University, Auckland, New Zealand
- Ellett, S. University of Auckland,; Nutrigenomics NZ, Auckland, New Zealand
- Ellison-Loschmann, L., Centre for Public Health Research, Massey University, Wellington, New Zealand
- Eyles, D., Queensland Brain Institute, University of Queensland, Brisbane, Australia
- Eyles, H., Faculty of Medical and Health Sciences, University of Auckland, Auckland, New Zealand
- Eyres, L., New Zealand Institute of Chemistry, Auckland, New Zealand
- Feng, P., Food Science, Faculty of Agriculture and Life Sciences, Lincoln University, Lincoln, Canterbury, New Zealand
- Ferguson, L., Faculty of Medical and Health Sciences, University of Auckland, Auckland, New Zealand
- Fleming, L., Department of Human Nutrition, University of Otago, Dunedin, New Zealand
- Firestone, R., Centre for Public Health Research, Massey University, Wellington, New Zealand
- Foliaki, S., Centre for Public Health Research, Massey University, Wellington, New Zealand
- Fong, B., Fonterra Co-operative Group, Palmerston North, New Zealand
- Forbes-Ewan, C., Defence Science and Technology Group, Tasmania, Australia
- Fraser, E., School of Food and Nutrition, Massey University, Auckland, New Zealand
- Fraser, K., AgResearch Grasslands, Palmerston North, New Zealand
- Gammon, C.S., School of Food and Nutrition, Massey University, Auckland, New Zealand
- Gearry, R., Christchurch District Health Board, Christchurch, New Zealand
- Gibbs, M., Food Science and Risk Assessment Directorate, Ministry for Primary Industries, Wellington, New Zealand Gibson, R.S., Department of Human Nutrition, University of Otago, Dunedin, New Zealand
- Grant, C.C., Centre for Longitudinal Research—He Ara ki Mua, The University of Auckland, Auckland, New Zealand
- Gris, F., School of Food and Nutrition, Massey University, Palmerston North, New Zealand
- Gunn, C., Gravida: National Centre for Growth and Development, University of Auckland, Auckland, New Zealand
- Gunn, J., Gravida: National Centre for Growth and Development, University of Auckland, Auckland, New Zealand
- Hampson, D.J., School of Veterinary and Life Sciences, Murdoch University, Murdoch, WA, Australia
- Harrex, H., Department of Human Nutrition, University of Otago, Dunedin, New Zealand
- Haslett, S.J., School of Food and Nutrition, Massey University, Palmerston North, New Zealand
- Haszard, J.J., Department of Human Nutrition, University of Otago, Dunedin, New Zealand
- Hawkins, R., Department of Human Nutrition, University of Otago, Dunedin, New Zealand
- Hawthorne, P., Food Science and Risk Assessment Directorate, Ministry for Primary Industries, Wellington, New Zealand
- Healey, G., Food, Nutrition and Health Group, Plant and Food Research Limited; School of Food and Nutrition, Massey University, Palmerston North, New Zealand
- Heath, A.-L.M., Department of Human Nutrition, University of Otago, Dunedin, New Zealand
- Hedderley, D., Nutrition and Health Group, Plant and Food Research Limited, Palmerston North, New Zealand
- Hempseed, G., Rowett Institute of Nutrition and Health, Aberdeen, United Kingdom
- Herath, T., School of Food and Nutrition, Massey University, Palmerston North, New Zealand
- Horwath, C., Department of Human Nutrition, University of Otago, Dunedin, New Zealand
- Houghton, L.A., Department of Human Nutrition, University of Otago, Dunedin, New Zealand
- Ingram, M.A., School of Food and Nutrition, Massey University, Auckland, New Zealand
- Jaquiery, A., Medical Research Council Lifecourse Epidemiology Unit, University of Southampton, Southampton, United Kingdom
- Jarrett, P., Department of Dermatology, Middlemore Hospital, Auckland, New Zealand
- Jayasinghe, S.N., School of Food and Nutrition, Massey University, Auckland, New Zealand
- Jesuthasan, A., Discipline of Nutrition and Dietetics, University of Auckland; Nutrigenomics NZ, Auckland, New Zealand
- Jia, L., School of Food and Nutrition, Massey University, Palmerston North, New Zealand
- Jin, Y., School of Food and Nutrition, Massey University, Palmerston North, New Zealand
- Jones, B., School of Food and Nutrition, Massey University, Auckland, New Zealand
- Jones, E., Department of Human Nutrition, University of Otago, Dunedin, New Zealand
- Kahn, D.E., Rowett Institute of Nutrition and Health, Aberdeen, United Kingdom
- Kaholokula, K., Department of Native Hawaiian Health, University of Hawaii, Manoa, Hawaii
- Kepa, M., General Practice and Primary Health Care, University of Auckland, Auckland, New Zealand
- Kerse, N., General Practice and Primary Health Care, University of Auckland, Auckland, New Zealand
- Kim, N., School of Public Health, Massey University, Wellington, New Zealand
- Kim, J.C., Department of Agriculture and Food WA, Perth, Australia
- Kruger, M., School of Food and Nutrition, Massey University, Palmerston North, New Zealand
- Kruger, R., School of Food and Nutrition, Massey University, Auckland, New Zealand
- Kruimer, D., Department of Human Nutrition, University of Otago, Dunedin, New Zealand
- Lagström, H., Turku Institute for Child and Youth Research, University of Turku, Turku, Finland
- Laing, B., Nutrition and Dietetics, University of Auckland; Nutrigenomics NZ, Auckland, New Zealand
- Lawrence, W., Medical Research Council Lifecourse Epidemiology Unit, University of Southampton, Southampton, United Kingdom
- Leong, S.L., Department of Human Nutrition, University of Otago, Dunedin, New Zealand
- Linderborg, K.M., Department of Biochemistry, University of Turku, Turku, Finland
- Ma, J., School of Food and Nutrition, Massey University, Palmerston North, New Zealand
- Ma, L., Fonterra Co-operative Group, Palmerston North, New Zealand
- Makela, J., Turku Institute for Child and Youth Research, University of Turku, Turku, Finland
- Mann, J.I., Department of Human Nutrition; Edgar National Centre for Diabetes and Obesity Research, University of Otago, Dunedin, New Zealand
- Mansur, F.A.F., Universiti Sains Islam Malaysia, Kuala Lumpur, Malaysia
- Manzor, N.F.M., Universiti Sains Islam Malaysia, Kuala Lumpur, Malaysia
- Markworth, J.F., Liggins Institute, University of Auckland, Auckland, New Zealand
- Marlow, G., Nutrition and Dietetics, University of Auckland; Nutrigenomics NZ, Auckland, New Zealand; Molecular Sciences, University of Cardiff, Cardiff, Wales
- Martell, S., Plant and Food Research, Palmerston North, New Zealand
- Martin, N., New Zealand Defence Force, Wellington, New Zealand
- Mason, S.L., Faculty of Agriculture and Life Sciences, Lincoln University, Lincoln, New Zealand
- Maxted, E., Dietetics, Northland District Health Board, Whangarei, New Zealand
- McClung, J., Military Nutrition Division, US Army Research of Environmental Medicine, Natick, USA
- McDonald, B., School of Food and Nutrition, Massey University, Auckland, New Zealand
- MacGibbon, A., Fonterra Co-operative Group, Palmerston North, New Zealand
- McJarrow, P., Fonterra Co-operative Group, Palmerston North, New Zealand
- McLean, R.M., Department of Human Nutrition, University of Otago, Dunedin, New Zealand
- Macluskie, G., Weipers Centre, Glasgow, United Kingdom
- McNab, M., Dietetic Services, Whangarei Hospital, Whangarei, New Zealand
- McNaughton, S.A., School of Exercise and Nutritional Sciences, Deakin University, Melbourne, Australia
- Mearns, G. School of Clinical Sciences, Auckland University of Technology, Auckland, New Zealand
- Meiklejohn, E., Department of Human Nutrition, University of Otago, Dunedin, New Zealand
- Mendes Borges, T., Faculty of Agriculture and Life Sciences, Lincoln University, Lincoln, New Zealand
- Meredith-Jones, K., Department of Medicine, University of Otago, Dunedin, New Zealand
- Milan, A.M., Liggins Institute, University of Auckland, Auckland, New Zealand
- Mirosa, M., Department of Food Science, University of Otago, Dunedin, New Zealand
- Moore, L., Boston University, Boston, United States
- Morris, A.C., Rowett Institute of Nutrition and Health, Aberdeen, United Kingdom
- Morton, H., School of Food and Nutrition, Massey University, Palmerston North, New Zealand
- Morton, J.D., Faculty of Agriculture and Life Sciences, Lincoln University, Lincoln, New Zealand
- Morton, S.M.B., Centre for Longitudinal Research—He Ara ki Mua, University of Auckland, Auckland, New Zealand
- Moyes, S, General Practice and Primary Health Care, University of Auckland, Auckland, New Zealand
- Muhlhausler, B. University of Adelaide, Adelaide, SA., Australia
- Ni Mhurchu, C., Faculty of Medical and Health Sciences, University of Auckland, Auckland, New Zealand
- Novotny, R., University of Hawaii at Manoa, Hawaii, United States
- Nowson, C. Deakin University, Geelong, VIC, Australia
- O'Brien, W.J., School of Food and Nutrition, Massey University, Auckland, New Zealand
- O’Connell, A., Child and Adolescent Mental Health Services, Capital & Coast District Health Board, Wellington, New Zealand
- Oey, I., Department of Food Science, University of Otago, Dunedin, Otago, New Zealand
- Parnell, W., Department of Human Nutrition, University of Otago, Dunedin, Otago, New Zealand
- Patil, S.S., Faculty of Agriculture and Life Sciences, Lincoln University, Lincoln, New Zealand
- Paturi, G., Plant and Food Research, Auckland, New Zealand
- Pedley, K.C., School of Food and Nutrition, Massey University, Palmerston North, New Zealand
- Pethick, D.W., School of Veterinary and Life Sciences, Murdoch University, Murdoch, WA, Australia
- Philipsen, S., School of Food and Nutrition, Massey University, Auckland, New Zealand
- Pileggi, C.A., Liggins Institute, University of Auckland, Auckland, New Zealand
- Pluske, J., School of Veterinary and Life Sciences, Murdoch University, Murdoch, WA, Australia
- Popovich, D., School of Food and Nutrition, Massey University, Palmerston North, New Zealand
- Poulsen, R., School of Food and Nutrition, Massey University, Palmerston North, New Zealand
- Prabha, S. UCSI University, Kuala Lumpur, Malaysia
- Pullar, J., Centre for Free Radical Research, University of Otago, Christchurch, New Zealand
- Pundir, S., Liggins Institute, University of Auckland, Auckland, New Zealand
- Quigg, R., University of Otago, Dunedin, New Zealand
- Rahim Elkahi, M.A.M.A., Universiti Sains Islam Malaysia, Kuala Lumpur, Malaysia
- Reynolds, A.N., Edgar National Centre for Diabetes and Obesity Research; Department of Human Nutrition, University of Otago, Dunedin, New Zealand
- Richter, M., School of Food and Nutrition, Massey University, Auckland, New Zealand
- Rivers, S., School of Food and Nutrition, Massey University, Auckland, New Zealand
- Rowan, A., Fonterra Co-operative Group, Palmerston North, New Zealand
- Roy, N.C., AgResearch Grasslands, Palmerston North; Riddet Institute, Massey University, Palmerston North, New Zealand; Gravida: National Centre for Growth and Development, University of Auckland, Auckland, New Zealand
- Ruan, W., Institute of Molecular and Cell Biology, Agency for Science, Technology and Research, Singapore
- Saeedi, P., Department of Human Nutrition, University of Otago, Dunedin, New Zealand
- Samman, S., Department of Human Nutrition, University of Otago, Dunedin, New Zealand
- Savage, G., Faculty of Agriculture and Life Sciences, Lincoln University, Lincoln, New Zealand
- Schlothauer, R., Comvita New Zealand Ltd., Paengaroa, New Zealand
- Schrijvers, J., School of Food and Nutrition, Massey University, Auckland, New Zealand
- Scragg, R., Faculty of Medical and Health Sciences, University of Auckland, Auckland, New Zealand
- Sergi, D., Rowett Institute of Nutrition and Health, Aberdeen, United Kingdom
- Share, A.N., School of Food and Nutrition, Massey University, Auckland, New Zealand; Dietetic Services, Whangarei Hospital, Whangarei, New Zealand
- Shields, E., Faculty of Medical and Health Sciences, University of Auckland, Auckland, New Zealand
- Shultz, S.P., School of Sport and Exercise, Massey University, Wellington, New Zealand
- Signal, L., Department of Public Health, University of Otago, Wellington South, New Zealand
- Skeaff, C.M, Department of Human Nutrition, University of Otago, Dunedin, New Zealand Skeaff, S.A., Department of Human Nutrition, University of Otago, Dunedin, New Zealand
- Skidmore, P.M.L., Department of Human Nutrition, University of Otago, Dunedin, New Zealand
- Slobbe, C., Department of Human Nutrition, University of Otago, Dunedin, New Zealand
- Smith, C., Department of Human Nutrition, University of Otago, Dunedin, New Zealand
- Smuts, C.M., Centre of Excellence for Nutrition, North-West University, Potchefstroom, North West, South Africa
- Song, H., Rowett Institute of Nutrition and Health, Aberdeen, United Kingdom
- Stoner, L., School of Sport and Exercise, Massey University, Wellington, New Zealand
- Stonehouse, W., Food and Nutrition Flagship, Commonwealth Scientific and Industrial Research Organisation (CSIRO), Adelaide, South Australia, Australia
- Tan, R.C.H. UCSI University, Kuala Lumpur, Malaysia
- Tannock, G., Department of Microbiology and Immunology, University of Otago, Dunedin, New Zealand
- Taylor, B.J., Edgar Diabetes and Obesity Research Centre, University of Otago; Department of Women’s and Children’s Health, Dunedin School of Medicine, University of Otago, Dunedin, New Zealand
- Taylor, R.W., Department of Medicine, University of Otago; Edgar Diabetes and Obesity Research Centre, University of Otago, Dunedin, New Zealand
- Teh, R., General Practice and Primary Health Care, University of Auckland, Auckland, New Zealand
- Thomson, C., Department of Human Nutrition, University of Otago, Dunedin, New Zealand
- Thomson, J.A., School of Food and Nutrition, Massey University, Palmerston North, New Zealand
- Thorstensen, E.B., Liggins Institute, University of Auckland, Auckland, New Zealand
- Ullah, I., School of Food and Nutrition, Massey University, Auckland, New Zealand
- Vanhanen, L., Faculty of Agriculture and Life Sciences, Lincoln University, Lincoln, New Zealand
- Venn, B., Department of Human Nutrition, University of Otago, Dunedin, New Zealand
- Vissers, M., Centre for Free Radical Research, University of Otago, Christchurch, New Zealand
- Vogel, C., Medical Research Council Lifecourse Epidemiology Unit, University of Southampton, Southampton, United Kingdom
- von Hurst, P.R., School of Food and Nutrition, Massey University, Auckland, New Zealand
- Wagner, K. Department of Nutritional Sciences, University of Vienna, Vienna, Austria
- Wall, C.R., Nutrition and Dietetics, University of Auckland, Auckland, New Zealand
- Wallace, A., Plant and Food Research Limited, Christchurch, New Zealand
- Walsh, D.C.I., Institute for Natural and Mathematical Sciences, Massey University, Auckland, New Zealand
- Weber, J.L., School of Food and Nutrition, Massey University, Palmerston North, New Zealand
- Webster, J., George Institute for Global Health, Sydney, Australia
- Wentzel-Viljoen, E., Centre of Excellence for Nutrition, North-West University, Potchefstroom, North West, South Africa
- Wham, C., School of Food and Nutrition, Massey University, Auckland, New Zealand
- Wheeler, B.J., Department of Women’s and Children’s Health, Dunedin School of Medicine, University of Otago, Dunedin, New Zealand
- Williams, L., Rowett Institute of Nutrition and Health, Aberdeen, United Kingdom
- Williams, S.M., Department of Preventive and Social Medicine, University of Otago, Dunedin, New Zealand
- Wong, J.E., Universiti Kebangsaan Malaysia, Kuala Lumpur, Malaysia
- Yan, X., Shanxi University of Traditional Chinese Medicine, Taiyuan City, Shanxi Province, China

